# Adipocyte Myoglobin Is a Determinant of Energy Expenditure and a Potential Target to Limit Obesity

**DOI:** 10.1002/advs.76191

**Published:** 2026-06-25

**Authors:** Christian Strehlau, Helen Broghammer, Claudia Gebhardt, Anne Hoffmann, Tobias Hagemann, Şeyma Midilli, Rachel S. Zimmer, Kristin Schubert, Vasiliki Karagiannakou, Anastasia Georgiadi, Mario Ost, Martin Krüger, Lisa Roth, Kerstin Krause, Nora Klöting, Maria Keller, Martin Wabitsch, Rima Nuwayhid, Roland H. Stimson, Michael Stumvoll, Matthias Blüher, Juliane Weiner, John T. Heiker

**Affiliations:** ^1^ Helmholtz Institute for Metabolic Obesity and Vascular Research (HI‐MAG) of the Helmholtz Zentrum München at the University of Leipzig and University Hospital Leipzig Leipzig Germany; ^2^ Cell Biology Faculty 2 – Environment and Natural Science Brandenburg Technical University Cottbus‐Senftenberg Senftenberg Germany; ^3^ Department of Molecular Systems Biology Helmholtz Centre for Environmental Research (UFZ) Leipzig Germany; ^4^ Institute For Diabetes and Cancer Helmholtz Diabetes Center Helmholtz Munich Neuherberg Germany; ^5^ German Center for Diabetes Research Neuherberg Germany; ^6^ Paul Flechsig Institute – Centre of Neuropathology and Brain Research University of Leipzig Leipzig Germany; ^7^ LeiCeM – Leipzig Center of Metabolism Leipzig University Leipzig Germany; ^8^ Institute of Anatomy Faculty of Medicine Leipzig University Leipzig Germany; ^9^ Department of Endocrinology Nephrology and Rheumatology Division of Endocrinology Leipzig University Medical Center Leipzig Germany; ^10^ Department of Pediatrics and Adolescent Medicine Division of Pediatric Endocrinology and Diabetes Ulm University Medical Center Ulm Ulm Germany; ^11^ Partner site Ulm German Center for Child and Adolescent Health (DZKJ) Ulm Germany; ^12^ Department of Orthopaedic Trauma and Plastic Surgery Division of Plastic Aesthetic and Special Hand Surgery University Hospital Leipzig Leipzig Germany; ^13^ Institute of Neuroscience and Cardiovascular Research University of Edinburgh Queen's Medical Research Institute Edinburgh UK; ^14^ Partner site Leipzig German Center for Child and Adolescent Health (DZKJ) Leipzig Germany; ^15^ Institute of Biochemistry Faculty of Life Sciences University of Leipzig Leipzig Germany

**Keywords:** brown adipose tissue, energy expenditure, lipid metabolism, metabolic disease, myoglobin, obesity, thermogenesis

## Abstract

Nutritional overflow and a positive energy balance are hallmarks of metabolic diseases including obesity and type 2 diabetes. Brown and beige adipocytes maintain systemic metabolic homeostasis by clearing and oxidizing energy‐rich nutrients during thermogenic activation. Myoglobin (MB) is classically regarded as a muscle‐associated oxygen‐binding protein; however it is also expressed in brown and beige adipocytes, where it contributes to intracellular lipid handling and oxidative metabolism. Here, we report that loss of MB exclusively in adipose tissue (AT) lowers whole‐body energy expenditure, impairs thermoregulation, and increases susceptibility to diet‐induced obesity. AT‐specific MB knockout (ATMBKO) mice exhibit elevated circulating triglycerides (TG) and fatty acids, indicating defective lipid clearance and utilization. Omics analyses reveal coordinated downregulation of oxidative phosphorylation, fatty acid metabolism, and myogenic programs. Conversely, restoration of MB in MB knockout (MBKO) mice improves metabolism in vivo. MB expression determines the capacity for mitochondrial fatty acid oxidation in brown adipocytes, whereas MB overexpression in primary human white adipocytes enhances thermogenic activity, confirming functional relevance of MB in human AT. Together, these findings establish MB as a key determinant of thermogenic lipid metabolism and energy expenditure in vivo and increasing adipocyte MB expression could increase energy expenditure and complement obesity treatment strategies.

## Graphical abstract

Myoglobin, known as a muscle oxygen‐carrying protein, is shown to play a key role in fat cells that burn energy. Loss of myoglobin reduces the body's ability to generate heat and increases obesity risk, while restoring it improves metabolism. The study identifies myoglobin as a regulator of fat burning and a potential target to enhance energy expenditure.

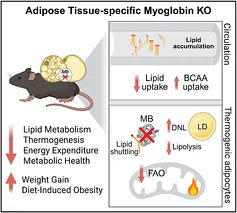

## 1 Introduction

Endothermal animals have the capacity to generate heat to maintain whole body temperature, thereby ensuring optimal metabolism [1]. Mammals developed non‐shivering thermogenesis in brown and beige adipocytes as an important pathway to defend the body against cold [1]. This is achieved through expression of the uncoupling protein 1 (UCP1), a proton transporter that locates in the inner mitochondrial membrane and uncouples oxidative phosphorylation from ATP synthesis. During cold exposure, increased sympathetic outflow leads to a multi‐organ response including activation of brown adipose tissue (BAT) [2]. The discovery of functional BAT in adult humans sparked a surge of interest in its role as a regulator of metabolic health [3, 4, 5].

When activated, BAT acts as a catabolic “sink” for circulating macro‐nutrients including glucose, lipids, and branched‐chain amino acids (BCAA). In humans, the presence of BAT and its activity are linked to improved body fat regulation [6], cardiometabolic health [7], lower odds of type 2 diabetes [6] and dyslipidemia [8]. Additionally, blood glucose and TG values are improved in humans with active BAT. These positive associations are more pronounced in individuals with overweight or obesity [7]. In mice, BAT activation leads to increased whole‐body energy expenditure, resistance to diet‐induced obesity [9], as well as improved glucose tolerance, insulin sensitivity [10], and lipid metabolism [9].

Lipid metabolism plays a central role in the thermogenic activity of brown and beige adipocytes. Adrenergic activation triggers free fatty acid (FFA) release from intracellular TG‐stores and uptake of lipids from the circulation to provide fuel for beta‐oxidation and mitochondrial respiration. Consequently, BAT activation corrects hyperlipidemia after short‐term cold exposure through clearance of TG‐rich lipoproteins from circulation [9]. Intracellular fatty acid binding proteins (FABP) solubilize the hydrophobic molecules acting as chaperones and shuttle systems for lipids between cellular organelles like mitochondria for beta‐oxidation, peroxisomes for processing, or lipid droplets for storage. Additionally, activated BAT releases specific lipids and metabolites, including 12‐HEPE [11] and 12,13 di‐HOME [12], that positively impact metabolism.

Recent studies have demonstrated MB expression in BAT [13, 14, 15, 16, 17] and established MB as a thermogenic protein in BAT biology [13]. There, its expression modulates intracellular lipid composition, lipid droplet morphology, and metabolic activity [13]. Similarly, substrate preference in cardiac muscle of MB‐deficient mice shifts from FFA to glucose oxidation [18] and cardiomyocytes display accumulation of TGs in intracellular lipid droplets [19]. Further, MB is ectopically expressed in mammary epithelial cells where it promotes FFA oxidation and reduces lipogenesis [20], and in mesenchymal stem cells where its presence is associated with increased FFA oxidation and regulation of pluripotency [21].

Higher levels of MB expression increase adrenergic activation, lipolysis, and mitochondrial respiratory activity of brown adipocytes [13]. Functional associations linking MB expression to lipid metabolism are supported by a growing body of evidence for MB lipid interaction. MB binds to long‐chain fatty acids (LCFA) [22, 23], medium‐chain fatty acids (MCFA) [24], and acylcarnitines [25] with varying affinity in the µm (Kd) range. Although the affinity of MB to FFAs is lower than that of other FABPs, MB provides high diffusivity in the cell and an oxygen‐dependent binding mechanism [26] distinguishing it from classical FABPs. Oxygenated MB specifically binds to lipids, whereas deoxygenated MB shows no or only unspecific binding [26, 27, 28]. This provides a feasible role for MB in cellular lipid trafficking. We recently demonstrated that MB acts as a lipid binding protein in brown adipocytes and that its beneficial properties for metabolism are dependent on the ability to bind lipids [13]. Upon activation in BAT, cytosolic lipid levels drastically rise, and MB could shuttle these lipids along an oxygen gradient to the sites of lipid metabolism releasing them together with oxygen.

A major drawback of current in vivo models is the whole‐body MBKO model, that disallows to distinguish whether AT expressed MB alone is responsible for metabolic alterations in these mice without confounding contributions from cardiac and skeletal muscle. Here, we demonstrate that loss of MB in AT alone lowers energy expenditure, resulting in impaired cold‐tolerance and increased diet‐induced obesity. Additionally, our data reveals dysregulation of lipid metabolism in mice lacking MB specifically in AT.

## 2 Results

### 2.1 ATMBKO Mice

**FIGURE 1:**
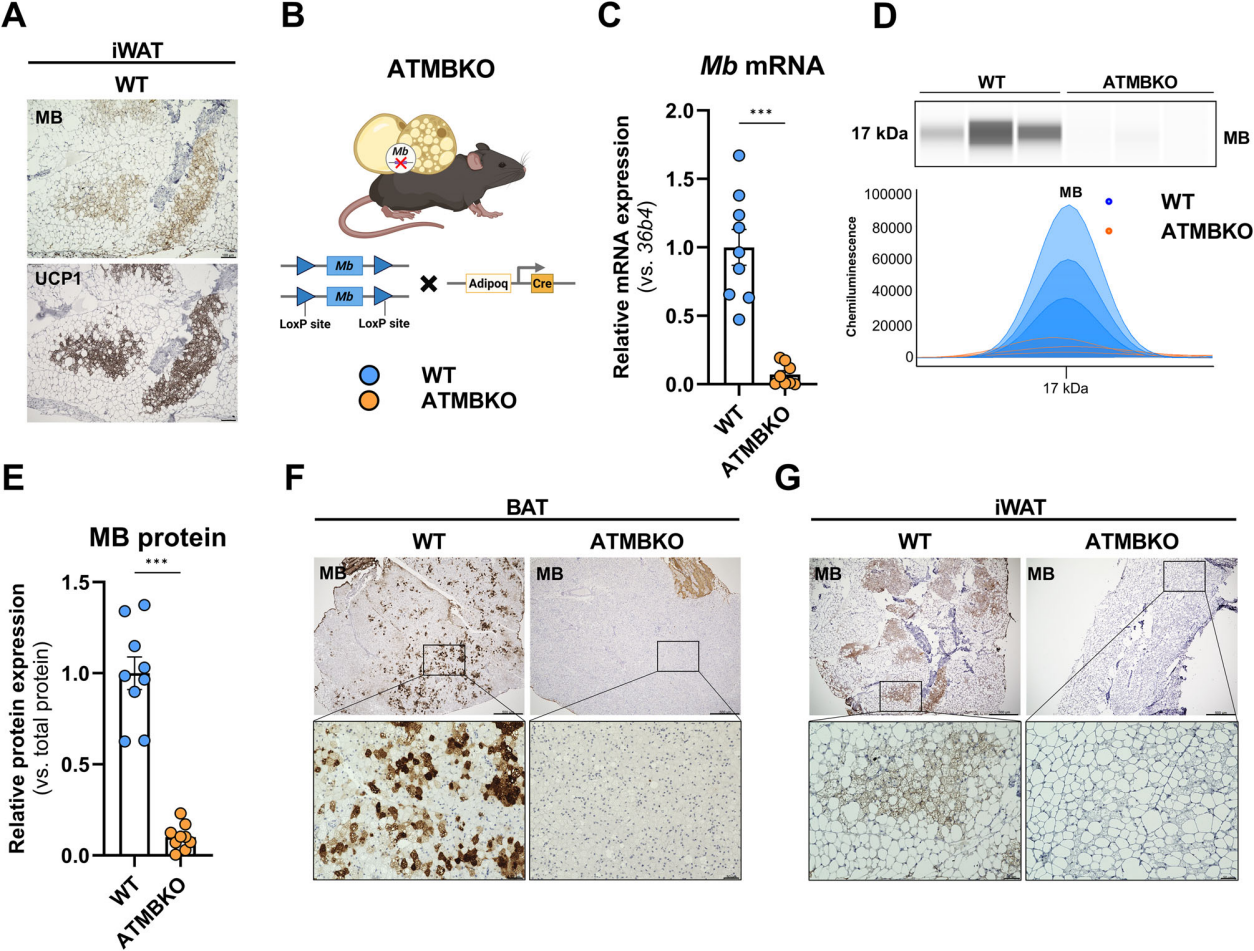
Generation of the ATMBKO mouse model. (A) Representative immunohistochemical staining of MB and UCP1 in iWAT of a cold‐acclimated female mouse showing co‐expression in beige adipocytes (scale bar: 500 µm for 4x, 100 µm for 10x and 50 µm for 20x magnification). (B) Schematic illustration of ATMBKO mouse model. Mice expressing Cre‐recombinase under control of the Adiponectin promoter (Adipoq‐Cre) were crossed with C57BL6/NTac mice with conditional (floxed) Mb alleles (Mb‐flox) to specifically delete Mb in adipocytes (created in BioRender). C) Relative Mb gene expression in BAT from cold‐acclimated female WT and ATMBKO mice (*n* = 9 per genotype). Created in BioRender, https://BioRender.com/5ts4hlf. (D) Representative capillary‐based immunoassays detecting MB protein levels in BAT from female WT and ATMBKO mice (*n* = 3 per genotype). (E) Quantification of MB protein levels in BAT from female WT and ATMBKO mice (*n* = 9 per genotype). (F,G) Representative immunohistochemical staining of MB in BAT (F) and iWAT (G) from female cold‐acclimated WT and ATMBKO mice (scale bar: 500 µm for 4x magnification and 50 µm for 20x magnification). Data are shown as mean ± SEM. Statistical significance is indicated by asterisks (^∗^ *p* < 0.05, ^∗∗^ *p* < 0.01, ^∗∗∗^ *p* < 0.001) and was determined by Student's t‐test (C, E).

Previous studies have demonstrated that MB is expressed in BAT of mice, especially after cold induced activation [13, 17, 20]. Whole‐body MB‐deficient mice displayed impaired thermoregulation with lower BAT surface temperature but did not allow to distinguish between the contribution of muscle MB and adipose MB to thermoregulation and energy expenditure. Chronic cold exposure leads to remodeling of white adipose tissue (WAT) with the appearance of thermogenic, multilocular adipocytes within the tissue [29, 30, 31]. Here, we show MB expression in multilocular and UCP1‐positive adipocytes of inguinal WAT (iWAT) sections from cold acclimated male wildtype (WT) mice (Figure 1A). Thus, we developed the first AT‐specific MB knockout mouse model aiming for deletion of MB in both brown and white adipocytes.

ATMBKO mice were generated by crossing *Mb*‐floxed mice and transgenic mice expressing the Cre recombinase under the control of the murine *adiponectin* promoter (*Adipoq*‐Cre, Figure 1B). ATMBKO mice had the Mb^flx/flx^_Cre^−/+^ genotype and Mb^flx/flx^_Cre^−/−^ littermates served as WT controls. Interscapular BAT from male and female mice housed at 8°C for 1 week showed high MB mRNA and protein expression in WT animals and no expression in ATMBKO animals (Figure 1C–E). Antibody staining for MB in BAT of male mice confirmed the tissue‐specific KO by staining MB in muscle tissue attached to the MB‐negative BAT (Figure 1F) and also, no MB antibody staining was detected in multilocular adipocytes within the iWAT of ATMBKO mice (Figure 1G). In skeletal and heart muscle, no differences in MB expression were detected between ATMBKO and WT animals (Figure S1A,B).

### 2.2 AT‐Specific MB Knockout Leads to Reduced Body Temperature and Energy Expenditure During Cold Challenge

**FIGURE 2:**
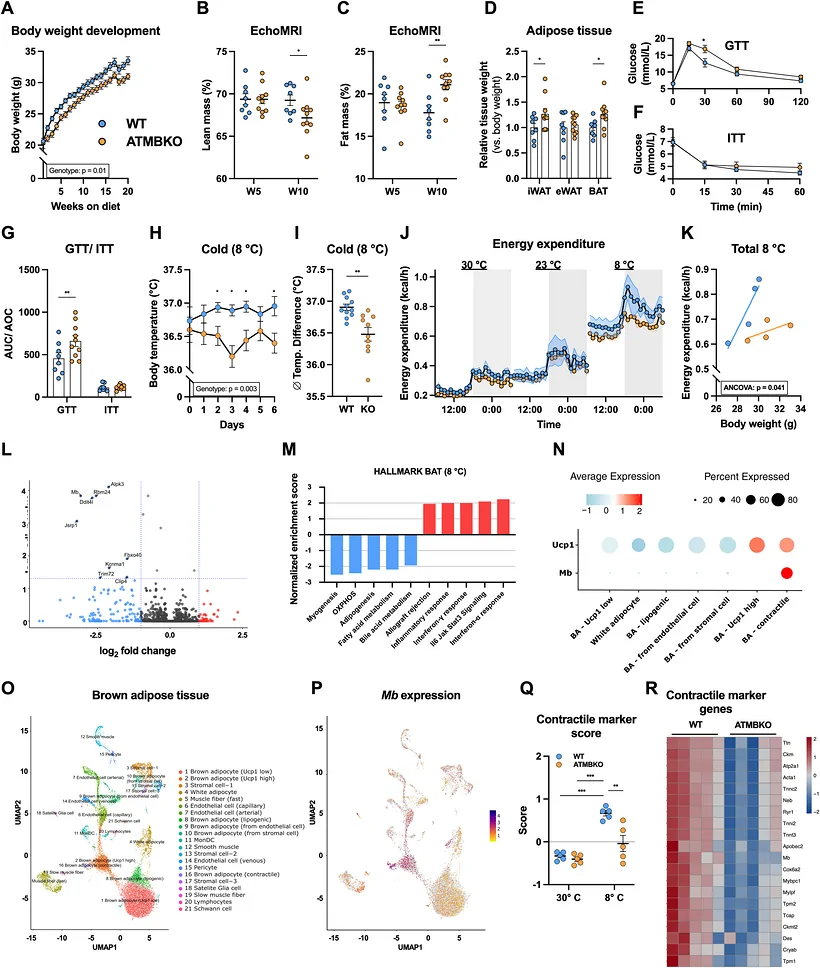
AT–specific MB knockout impairs thermoregulation and energy expenditure during chronic cold exposure. (A) Body weight development of male chow‐fed WT and ATMBKO mice from 5 to 24 weeks of age (*n* = 8/10). (B‐C) Percentage of lean (B) and fat mass (C) as determined by EchoMRI at 10 (week 5 on the HFD) and 16 (week 10 on the diet) weeks of age in male WT and ATMBKO mice (*n* = 8/10). (D) Relative weights of different AT depots at 24 weeks of age in male WT and ATMBKO mice (*n* = 8/10; relative to WT). (E‐G) Blood glucose levels during (E) glucose (GTT) and (F) insulin (ITT) tolerance tests and (G) area under (GTT) and over (ITT) the curve calculations (n = 8/10). (H‐I) Rectal body temperature of cold‐acclimated male and female WT and ATMBKO mice (8 °C for 1 week, *n* = 7/6 for female, *n* = 4/4 for male). (J,K) Male WT and ATMBKO mice were housed in metabolic cages for 5 days each at RT (23 °C), thermoneutrality (30 °C), and in the cold (8 °C). (J) Energy expenditure data is presented as an average of 3 consecutive days. (K) Regression plot of body weight with total (24 h) energy expenditure of cold‐exposed WT and ATMBKO mice (*n* = 4 per genotype). (L,M) Bulk RNA‐Seq data were obtained from BAT of male WT and ATMBKO mice housed at either thermoneutrality or cold for 24 h (*n* = 5 per genotype and temperature). (L) Volcano Plot showing differentially expressed genes (DEG) and (M) Gene Set Enrichment Analysis (GSEA) of pre‐ranked gene lists showing the top 5 up‐ and downregulated pathways from Hallmark gene sets in cold‐exposed ATMBKO mice compared to WT littermates. (N) Mb expression in different brown adipocyte subclusters determined by single nuclei RNA sequencing of BAT from WT mice [41]. (O) UMAP of single nuclei RNA sequencing data from BAT of WT mice housed at RT, as well as after acute and chronic cold exposure depicting different cell types within the BAT. (P) UMAP of single nuclei RNA sequencing data depicting Mb expression in different cell types within BAT. (Q) Expression score for contractile brown adipocyte marker expression in BAT of male ATMBKO and WT mice housed at 30°C or exposed to 8°C (*n* = 5/5 per temperature). (R) Heatmap depicting relative gene expression of top 20 marker genes uniquely expressed in contractile brown adipocytes in BAT of male ATMBKO and WT mice exposed to 8°C (*n* = 5/5). Data are shown as mean ± SEM. Statistical significance is indicated by asterisks (^∗^ *p* < 0.05, ^∗∗^ *p* < 0.01, ^∗∗∗^ *p* < 0.001) and was determined by Student's t test (G, I), two‐way ANOVA with Šidák post hoc test (A, B, C, Q) or Fisher's LSD test (D, H) and ANCOVA performed by CalR (K).

Surprisingly, male ATMBKO mice receiving a chow diet gained significantly less body weight, although the difference was < 5% at 12 and 20 weeks of age (Figure 2A and Figure S1C). However, body composition revealed a shift to proportionally higher fat and lower lean mass in ATMBKO mice compared to WT littermates (Figure 2B,C). The increase in body fat was driven by relatively higher iWAT and BAT weights in chow fed 24‐week‐old ATMBKO mice compared to WT littermates (Figure 2D). Additionally, ATMBKO mice exhibited significantly impaired glucose tolerance (Figure 2E,G) without changes in insulin sensitivity (Figure 2F,G). Together, these data demonstrate that loss of MB in AT affects body composition and promotes a metabolically impaired phenotype already under chow conditions.

To determine the contribution of adipose MB on thermogenesis and thermoregulation, we exposed 12‐week‐old chow‐fed mice to chronic cold (8°C) for 1 week. Both male and female ATMBKO mice showed significantly lower body temperatures (Figure 2H and Figure S1D,E for separated data) compared to WT littermates with an average difference of ∼0.5°C (Figure 2I) and without changes in food intake (Figure S1F). This is consistent with our previous studies in whole‐body MB‐KO mice that revealed impaired thermoregulation with reduced body temperature at temperatures below thermoneutrality [13]. Indirect calorimetry of male mice subsequently housed at room temperature (23°C), at thermoneutrality (30°C) and in the cold (8°C) revealed that ATMBKO mice exhibit significantly lower energy expenditure in the cold when BAT is fully active, both during the active (dark) and inactive (light) phase (Figure 2J,K) and without changes in locomotor activity or food intake (Figure S1G,H) compared to WT littermates. Also, expression of genes facilitating muscle thermogenesis were not different in skeletal muscle of ATMBKO and WT mice after 5 days of cold (8°C) adaptation (Figure S1A). At room temperature and at thermoneutrality, no differences were observed in energy expenditure (Figure 2J), food intake, and locomotor activity between both genotypes (Figure S1I–L).

To better understand molecular changes associated with MB loss in AT, we performed bulk RNA sequencing (RNA‐seq) analyses of intrscapular BAT from male mice housed either at thermoneutrality or in the cold for 24 h, thereby inactivating or activating BAT, respectively. Principal component analysis displayed clear separation of BAT transcriptomes between ATMBKO and WT littermates after short term cold exposure but not at thermoneutrality, in line with the role of MB in BAT metabolism (Figure S1M). Analysis of differentially expressed genes (DEGs) from ATMBKO against WT littermates identified nine significantly downregulated genes (*Mb*, *Rbm24*, *Kcnma1*, *Trim72*, *Ddit4l*, *Alpk3*, *Jsrp1*, *Fbxo40*, *Clip4*) after adjusting for multiple testing, among them MB as the top downregulated transcript (Figure 2L and Tables S1 and S2). All DEGs are primarily described in muscle function. *Rbm24* and *Ddit4l* are expressed in cardiomyocytes in response to stress. *Rbm24* regulates lipid metabolism and controls cell fate decisions [32] while *Ddit4l* is a mTOR inhibitor modulating metabolism and mitochondrial dynamics [33, 34]. *Kcnma1* [35, 36], *Trim72* [37, 38, 39] and *Jsrp1* are involved in muscle calcium signaling. *Trim72* and *Fboxo40* are further associated with insulin signaling [40]. To obtain insight into more subtle transcriptional changes we performed gene set enrichment analysis (Table S3). This revealed myogenesis, oxidative phosphorylation, adipogenesis, fatty acid metabolism, and bile acid metabolism as the most downregulated pathways in BAT of ATMBKO mice after cold exposure (Figure 2M). Upregulated pathways were linked to immune responses. Leveraging publicly available single nuclei RNA sequencing data of BAT from mice housed at room temperature or exposed to chronic or acute cold [41] (Figure 2N), we identified that MB is a marker gene of a brown adipocyte subcluster that is UCP1‐positive and defined by high expression of muscle‐specific and oxidative phosphorylation genes termed contractile brown adipocytes (Figure 2O,P and Figure S1N–P). Brown adipocytes have muscle‐like origins [30], express muscle‐specific genes [42], and their mitochondrial proteome and transcriptome resemble that of muscle mitochondria [43]. Further, contractile processes in UCP1‐positive brown adipocytes are involved in BAT activation and oxidative metabolism [44]. We observe that genes defining the muscle‐enriched (contractile) brown adipocyte subcluster are significantly downregulated in BAT of cold‐exposed ATMBKO mice compared to WT (Figure 2Q,R), suggesting that MB is necessary to maintain this oxidative brown adipocyte subcluster.

These data confirm the key role of AT expressed MB in thermogenesis and metabolism during BAT activation and demonstrates that loss of MB in AT alone is sufficient to lower body temperature and energy expenditure especially during cold exposure, and this is driven by metabolic changes with impaired fatty acid metabolism in BAT. Additionally, MB is an important factor to maintain a contractile brown adipocyte subpopulation.

### 2.3 Lower Energy Expenditure Exacerbates Diet‐Induced Obesity in ATMBKO Mice

The thermogenic activity of BAT is increased in response to high caloric diets to waste excess energy and limit body weight gain [30, 45]. Accordingly, energy expenditure is adaptively increased upon high‐fat diet (HFD) feeding when compared to normal chow diets [46]. Therefore, we examined whether loss of MB in AT promotes weight gain in mice when exposed to a HFD.

**FIGURE 3:**
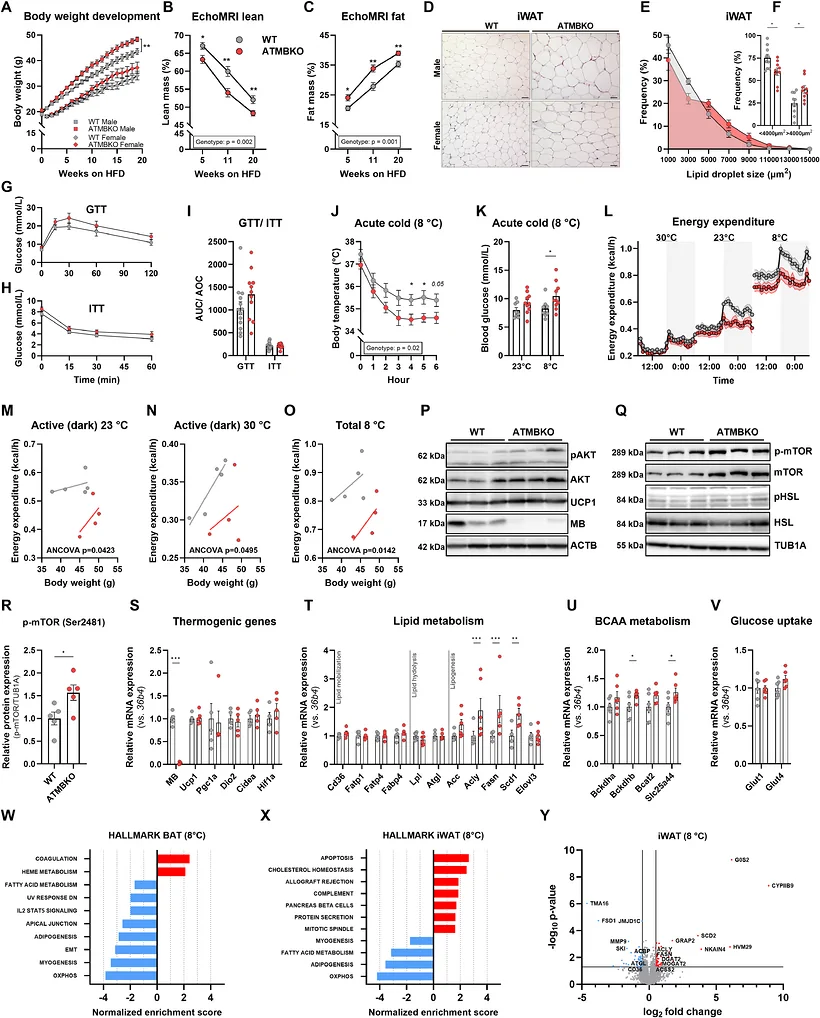
AT–specific MB knockout promotes diet‐induced obesity by reducing energy expenditure. (A) Body weight development of male and female HFD‐fed WT and ATMBKO mice from 5 to 24 weeks of age. Diet started at 5 weeks of age (*n* = 11/9 for female, *n* = 18/19 for male). (B,C) Percentage of lean (B) and fat mass (C) as determined by EchoMRI after 5, 11, and 20 weeks on a HFD in male and female WT and ATMBKO mice (*n* = 12/12 for female, *n* = 19/20 for male). (D) Representative H&E staining of iWAT from male and female HFD‐fed WT and ATMBKO mice (20x magnification, scale bar = 200 µm). (E) Relative frequency of adipocyte size (µm^2^) and (F) comparison of adipocyte frequencies <4000 and >4000 µm^2^ in iWAT of HFD‐fed male and female WT and ATMBKO mice (*n* = 5/5 for female, *n* = 5/4 for male). In (E), adipocyte sizes were assigned into 2000 µm^2^ bins. For frequency calculation, data points show bin centers. (G–I) Intraperitoneal glucose (G) and insulin (H) tolerance tests (GTT at 22 weeks and ITT at 23 weeks of age) and areas under/ over the curve (I) in female and male HFD‐fed WT and ATMBKO mice (GTT: *n* = 8/6 for female, *n* = 5/5 for male; ITT: *n* = 9/7 for female, *n* = 5/5 for male). (J,K) Female and male WT and ATMBKO mice on HFD were fasted (2 h pre cold exposure) and exposed to acute cold (8 °C) (*n* = 5/4 for female, *n* = 3/5 for male). Rectal body temperature (J) and blood glucose levels (K) were measured. (L–N) Male WT and ATMBKO mice housed in metabolic cages at RT (23 °C), thermoneutrality (30 °C), and chronic cold (8 °C). Data represent the average over 3 consecutive days (*n* = 5/4). (M) Regression plot of body weight with nighttime (12 h) energy expenditure at 23°C and (N) 30°C and (O) with total (24 h) energy expenditure at 8°C. (P,Q) Representative Western blot analysis of (P) pAKT/AKT, UCP1, MB, ACTB, and (Q) p‐mTOR/mTOR, pHSL/HSL, TUB1A expression in BAT of HFD‐fed WT and ATMBKO littermates after 5 days of cold exposure. (R) Relative p‐mTOR signal normalized by TUB1A after densitometric quantification (*n* = 5 per genotype). (S–V) mRNA expression of genes involved in (S) thermogenesis (*n* = 5 per genotype) (T) lipid metabolism (*n* = 5/ 6), (U) BCAA metabolism (*n* = 6 per genotype) and (V) glucose uptake (*n* = 6 per genotype) in BAT from HFD‐fed WT and ATMBKO mice after 5 days of cold exposure. Gene expression is normalized to 36b4 and relative to WT littermates. (W) GSEA of proteomics data from (W) BAT and (X) iWAT of male HFD‐fed WT and ATMBKO mice after 5 days cold exposure. Depicted are all significantly regulated pathways from the Hallmark gene set. (Y) Volcano plot showing significantly regulated proteins in iWAT of HFD‐fed and cold exposed (5 days) ATMBKO mice compared to WT littermates. Data are shown as mean ± SEM. Statistical significance is indicated by asterisks (^∗^ *p* < 0.05, ^∗∗^ *p* < 0.01) and was determined by two‐way ANOVA (A) and with Fisher's LSD test (B‐C, J‐K, S‐T), multiple t‐test (U), and Student's t‐tests (R) and ANCOVA performed by CalR (M, N, O).

Male and female ATMBKO and WT littermates were fed a HFD for 20 weeks and both sexes exhibited significant phenotypic differences. ATMBKO mice gained significantly more weight compared to WT littermates, with a final increase in body weight of approximately 10% in both male and female mice (Figure 3A). The increase in body weight went along with proportionally higher fat and lower lean mass after 5, 11, and 20 weeks of HFD feeding (Figure 3B,C and Figure S2A–D for separated data). Importantly, absolute lean mass was not different after 20 weeks on the HFD in both male and female ATMBKO mice compared to WT littermates (Figure S2B,D). The mass of different fat depots was significantly increased for iWAT only in male ATMBKO mice (Figure S2E,F). However, the analysis of adipocyte size from iWAT and epididymal WAT (eWAT) of both male and female mice revealed significant adipocyte hypertrophy in ATMBKO mice (Figure 3D–F and Figure S2G–K).

To assess consequences of diet‐induced obesity on metabolic health of the animals, we performed glucose (GTT) and insulin (ITT) tolerance test in ATMBKO and WT littermates after HFD feeding. Blood glucose levels in male ATMBKO mice were higher compared to WT littermates at week 20 of diet (Figure S2L). Also, glucose tolerance was lower in combined analysis of GTTs from male and female mice (Figure 3G,I and Figure S2M,N for separated data) but without reaching statistical significance. Insulin sensitivity, however, showed no difference albeit higher fasting glucose levels in ATMBKO animals of both sexes (Figure 3H,I and Figure S2O,P for separated data).

Since HFD feeding fundamentally alters substrate utilization, lipid storage and mobilization in AT, we aimed to investigate the functional consequences of MB deficiency for thermogenic capacity during fasting and cold exposure after HFD feeding. ATMBKO mice that were exposed to short‐term cold (acute cold, without food) exhibited significantly lower core body temperature compared to WT littermates with a difference of 1°C after 4–6 h in the cold (Figure 3J and Figure S2Q,R for separated data), and blood glucose levels were significantly higher in ATMBKO animals (Figure 3K). Thermogenesis in BAT during acute cold exposure heavily relies on the presence of exogenous substrates provided by WAT lipolysis, especially in the fasted state [47, 48]. Similarly, during overnight fasting BAT thermogenesis relies on available substrates from the circulation [49, 50]. Here, female AMTBKO mice exhibited significantly lower body temperature after 16 h of fasting (at 23°C) than WT littermates (Figure S2S).

Next, we examined whole body energy expenditure by indirect calorimetry in male ATMBKO and WT animals after 18 weeks of HFD feeding. Here, ATMBKO mice displayed significantly lower energy expenditure during the active (feeding) phase already at 23°C and 30°C (Figure 3L–N and Figure S2T,U). In WT animals, HFD feeding increased energy expenditure compared to chow, especially during the active feeding phase (Figures 2J and 3L). This strong increase is absent in ATMBKO animals, suggesting reduced postprandial BAT thermogenesis, as locomotor activity and food intake were not different (Figure S2V,W). Consistently, total energy expenditure is strongly reduced during chronic cold exposure in ATMBKO mice (Figure 3L,O and Figure S2X). Again, gene expression analysis revealed no sign for compensatory muscle thermogenesis in skeletal muscle of ATMBKO animals compared to WT littermates (Figure S2Y).

To observe regulatory changes in BAT signaling between ATMBKO and WT littermates, we next examined phosphorylation of key enzymes controlling BAT function. Phosphorylation of AKT, HSL, and PKA was unchanged after 5 days of cold exposure (Figure 3P,Q and Figure S2Z) indicating no changes in adrenergic tone and lipolysis. Phosphorylation of mTOR at Ser2481 on the other hand was markedly induced in animals lacking MB in AT (Figure 3Q,R). Further, mRNA levels of brown marker genes (*Ucp1*, *Ppargc1a*, *Dio2*) were unchanged (Figure 3S) but genes involved in de‐novo lipogenesis (*Acly*, *Fasn*), lipid saturation (*Scd1*) and BCAA metabolism were significantly higher in ATMBKO animals (Figure 3T,U). Expression of glucose transporters (*Glut1*, *Glut4*) were unchanged between both genotypes (Figure 3V).

To gain additional insight on molecular changes underlying the metabolic phenotype of ATMBKO mice, we performed untargeted proteomics analysis of BAT and iWAT from HFD‐fed WT and ATMBKO animals exposed to cold (8°C, males; Tables S4 and S5) or housed at RT (23°C, females; Tables S6 and S7). Global proteome profiling revealed downregulation of oxidative phosphorylation, fatty acid metabolism, myogenesis, and adipogenesis pathways in both adipose depots lacking MB and independent of housing temperature or sex respectively (Figure 3W,X and Figure S3A,B and Table S8). Among the top differentially abundant proteins in BAT from both temperatures and iWAT from RT are only few thermogenic or lipid metabolism markers (Figure S3C–E). However, the iWAT of cold exposed and HFD‐fed male ATMBKO shows changes in the expression of many classical markers of lipid metabolism (Figure 3Y). The most significantly upregulated protein is G0S2 (log2FC ∼6.2), an inhibitor of ATGL [51], and ATGL itself is significantly downregulated indicating reduced lipolytic activity. In addition, several enzymes involved in lipid synthesis were significantly upregulated, including ACLY and ACSS2 (generation of acetyl‐CoA), FASN (de novo fatty acid synthesis), MOGAT2 (conversion of MAG to DAG), and DGAT2 (conversion of DAG to TAG), indicating increased lipogenic activity. The significantly lower abundance of JMJD1C [52] may reflect impaired adipogenesis further driving adipocyte hypertrophy in diet‐induced obesity. These changes in the proteome of iWAT from AMTBKO mice clearly support the observed morphologic differences and phenotypic changes including adipocyte hypertrophy and increased iWAT tissue mass.

Together, these findings indicate that loss of adipocyte MB reduces energy expenditure and thermogenic capacity, promoting diet‐induced obesity and driving a metabolic shift toward increased lipid storage and impaired lipid utilization in both BAT and iWAT.

### 2.4 Loss of MB in AT Alters Whole Body Substrate Utilization

**FIGURE 4:**
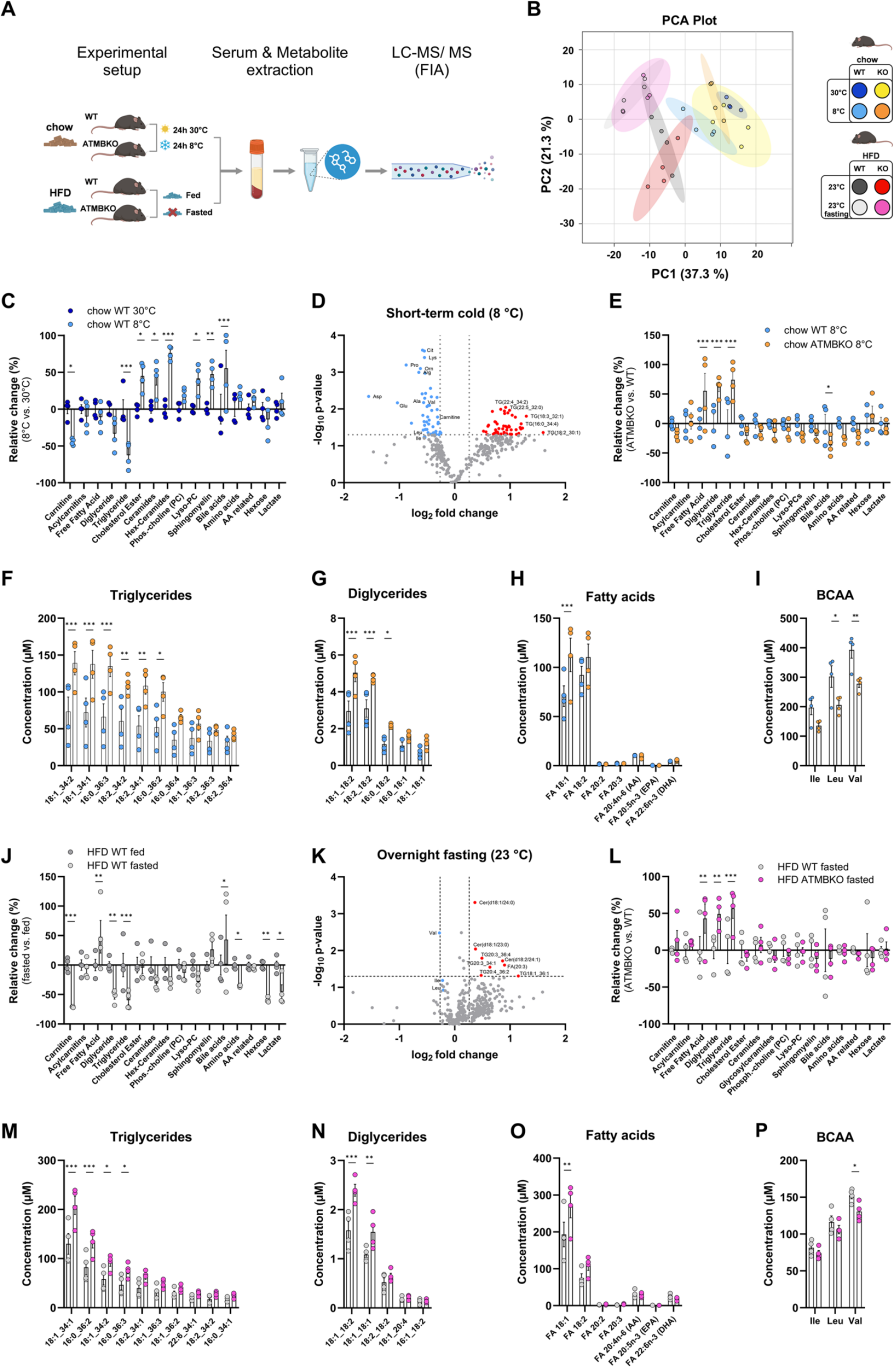
Targeted metabolomics indicates impaired lipid and compensatory BCAA metabolism in ATMBKO animals during metabolic stress. (A) Targeted metabolomics in arterial serum from WT and ATMBKO mice. Created in BioRender, https://BioRender.com/5ts4hlf. (B) Principal component analysis showing differences in metabolite abundance between WT and ATMBKO mice in chow‐fed male mice (1) housed at thermoneutrality (30°C) or (2) acutely exposed to cold (8°C for 24 h), as well as in female HFD‐fed mice housed at room temperature (RT, 23°C) (3) fed ad libitum or (4) fasted overnight for 16 h (*n* = 4 for all groups and genotypes). (C) Relative abundance of measured metabolite classes in male chow‐fed WT mice housed in the cold (8°C for 24 h) compared to thermoneutrality (30°C). (D) Volcano plot depicting changes in metabolites in serum of male ATMBKO compared to WT mice after short‐term cold exposure (8°C for 24 h, *n* = 4 per genotype). (E) Relative abundance of measured metabolite classes in male chow‐fed ATMBKO compared to WT mice after short‐term cold exposure (8°C for 24 h). (F‐I) Absolut concentrations of (F) the 10 most abundant TGs, (G) the 5 most abundant DGs, (H) measured FFAs, and (I) BCAAs isoleucine, leucine, and valine. (J) Relative abundance of measured metabolite classes in female HFD‐fed WT mice housed at RT (23°C) and fasted overnight (16 h) compared to WT mice fed ad libitum. (K) Volcano plot depicting changes in metabolites in serum of female HFD‐fed ATMBKO compared to WT mice after an overnight fast (16 h at 23°C, *n* = 4 per genotype). (L) Relative abundance of measured metabolite classes in female HFD‐fed ATMBKO compared to WT mice after an overnight fast (16 h at 23°C, *n* = 4). (M‐P) Absolute concentration of (M) the 10 most abundant TGs, (N) 5 most abundant DGs, (O) measured FFAs, and (P) BCAAs isoleucine, leucine, and valine. Data are shown as mean ± SEM. Statistical significance is indicated by asterisks (^∗^ *p* < 0.05, ^∗∗^ *p* < 0.01, ^∗∗∗^ *p* < 0.001) and was determined by two‐way ANOVA with Fisher's LSD test (C, E, J, L) or with Šidák post hoc test (F‐I, M‐P).

ATMBKO mice displayed impaired metabolism leading to increased body weight and body fat as well as blood glucose levels and glucose tolerance, both on chow and HFD. Additionally, thermoregulation was impaired in ATMBKO mice. Cold exposure has dramatic effects on systemic metabolism and fuel utilization to maintain body temperature. Since ATMBKO lead to reduced energy expenditure, especially in the cold, we aimed to understand the metabolic changes induced by loss of MB in AT in more detail. We performed targeted metabolomics (Biocrates MxP Quant 500) on arterial blood serum of male chow fed mice housed at thermoneutrality or in the cold for 24 h. Additionally, female HFD‐fed ATMBKO mice fasted overnight displayed significantly reduced body temperature after fasting, indicating impaired metabolism and thermoregulation. Therefore, targeted metabolomics was also performed on serum of female HFD‐fed mice housed at 23°C fed ad libitum or fasted overnight (Figure 4A).

Principal component analysis of all 8 groups reveals that the serum metabolome is mostly separated by diet and intervention (cold, overnight fasting). Separation between genotypes is especially visible for 24 h cold exposure in chow fed animals (Figure 4B).

In WT mice, short‐term cold exposure induced substantial changes in serum metabolites of chow fed male mice when compared to littermates housed at thermoneutrality (Figure 4C). Serum TG concentrations dropped by ∼60% accompanied by a reduction of carnitine levels by ∼55%, indicating that TGs are efficiently used for thermogenesis. Amino acids (AA) and AA related metabolites showed smaller reductions after cold. Additionally, concentrations of cholesterol esters, ceramides, glycosylceramides, sphingomyelins, and bile acids were significantly higher in mice after short‐term cold exposure (Figure 4C). Volcano plots displaying high and low abundant metabolites in cold exposed male chow‐fed (Figure 4D) ATMBKO mice versus WT littermates reveal that especially TGs are enriched, and AAs are decreased in the serum of ATMBKO mice. Overall abundance of metabolite classes revealed that TG, diglyceride (DG), and FFA concentrations were significantly higher in serum of ATMBKO mice exposed to cold (Figure 4E–H), while bile acids were significantly reduced (Figure 4E and Figure S4A,B). This is consistent with BAT transcriptomics data where fatty acid and bile acid metabolism were among the most downregulated pathways in ATMBKO mice (Figure 2M). Further, AAs (AA) concentrations were reduced (Figure 4E). Especially BCAAs are effectively catabolized by active BAT, and these were significantly lower (Figure 4I) in cold‐exposed ATMBKO animals compared to WT littermates. At thermoneutrality, abundance of most metabolites is not different between ATMBKO and WT littermates (Figure S4C) except for increased FFA concentrations in ATMBKO mice (Figure S4D).

HFD feeding led to significantly higher FFA and ceramide concentrations in the circulation of ATMBKO mice after 20 weeks of diet (Figure S4E,F). Although, these samples were obtained from mice during the inactive phase where energy expenditure was not different between the genotypes. Postprandial metabolite abundance could be more effective to detect changes in substrate clearance in HFD fed ATMBKO mice. However, overnight fasting in these mice revealed similar impairments as for male cold exposed and chow fed mice. In WT mice, fasting significantly reduced TG, DG, and carnitine concentrations while FFAs were higher abundant compared to fed WT mice. Further, AA concentrations as well as hexose and lactate levels were significantly reduced after fasting (Figure 4J). Volcano plot of fasted ATMBKO versus WT littermates shows higher abundance of TGs and ceramides while Valine is the most decreased metabolite (Figure 4K). Again, TG, DG, and FFA concentrations were significantly higher (Figure 4L–O) and BCAA levels were significantly lower (Figure 4P) in serum of fasted ATMBKO mice compared to WT littermates. We further analyzed serum of female HFD fed mice exposed to acute cold (6 h) without access to food. There, FFA levels were elevated in ATMBKO (Figure S4G,H) and again a marked reduction in BCAA levels was detected (Figure S4I) along with reduced lactate levels in ATMBKO compared to WT (Figure S4J).

In summary, serum metabolome data uncover a universal dysregulation of lipid clearance in both cold exposed male chow‐ and fasted female HFD‐fed ATMBKO mice. Especially upon activation of BAT (short‐term cold, overnight fasting), TG, DG and FFA levels remain significantly higher in serum of ATMBKO mice while BCAA levels are reduced. These data indicate that upon loss of MB in AT, lipid utilization for thermogenesis is impaired and to compensate, more AAs and especially BCAAs are taken up to meet the increased fuel demand. Notably, gene expression of key enzymes (*Bcat2, Bckdha, Bckdhb*) of BCAA metabolism and BCAA transporter *Slc25a44* were increased in BAT of male ATMBKO mice (Figure 2R).

### 2.5 MB Drives Mitochondrial Fatty Acid Oxidation

The increased retention of energy‐rich lipids in the serum of ATMBKO animals led us to analyze the dependence of lipid oxidation and uptake on MB in brown adipocytes in more detail. We have previously demonstrated that MB‐mediated lipid binding in brown adipocytes is underlying the MB‐driven increase in mitochondrial respiratory capacity [13]. Here, we adapted the LCFA oxidation stress test for the seahorse analyzer to investigate the impact of MB expression on the ability to use various exogenous fatty acids as fuel for mitochondrial respiration.

**FIGURE 5:**
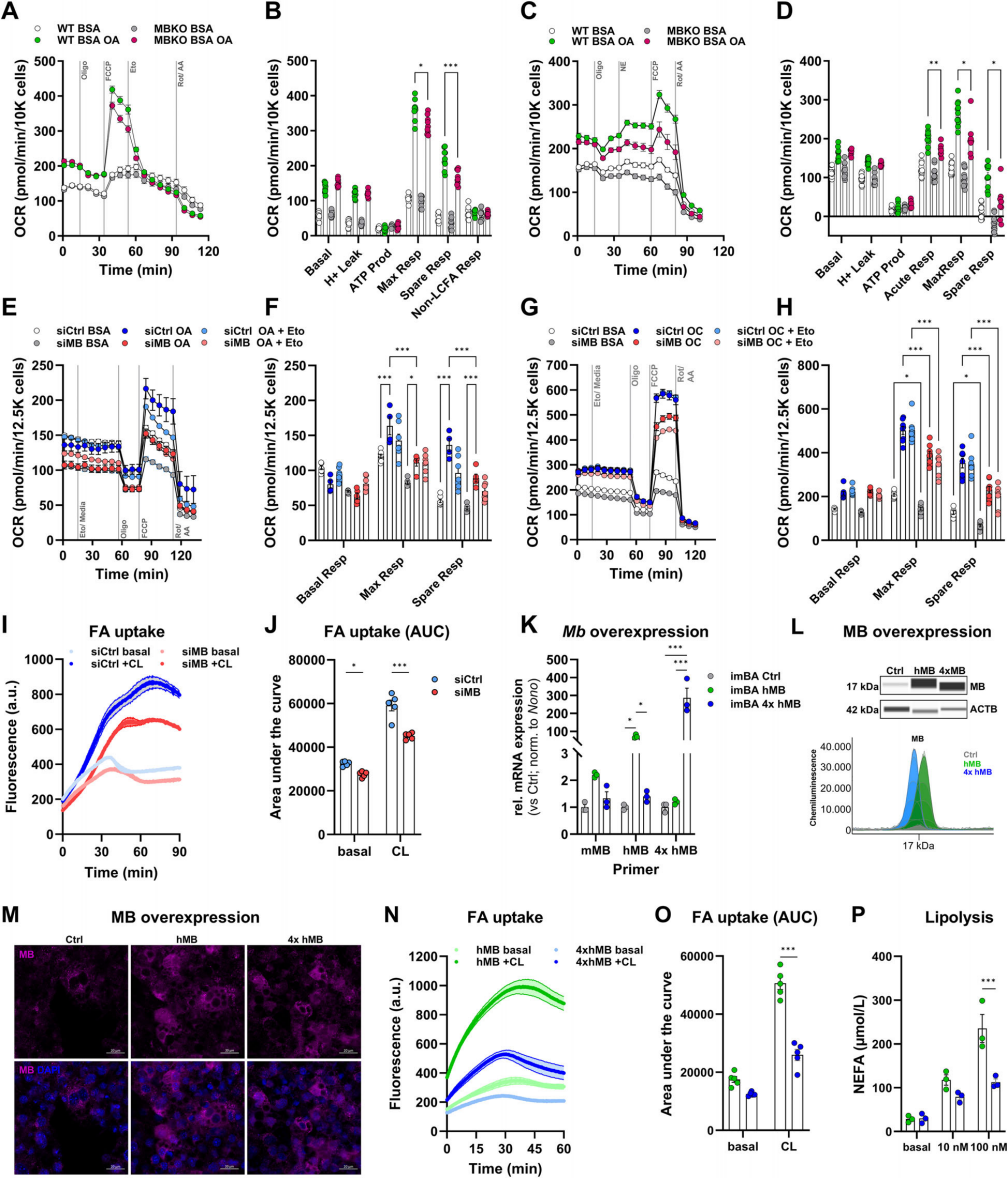
MB is rate‐limiting for mitochondrial fatty acid oxidation and cellular fatty acid handling. (A–D) Seahorse measurements of oxygen consumption rate (OCR) in fully differentiated primary brown adipocytes from MBKO and WT mice. (A) Mitochondrial FFA oxidation stress test for BSA‐conjugated oleate (OA) with etomoxir (Eto) injection after uncoupling and (B) quantification of basal respiration (resp.), proton leak, ATP production, maximal respiration, spare respiratory capacity and non‐LCFA respiration in brown adipocytes from MBKO and WT mice. (C) Mitochondrial FFA oxidation stress test for OA with acute injection of norepinephrine (NE) before uncoupling and (D) quantification of basal respiration, proton leak, ATP production, maximal respiration, spare respiratory capacity, and non‐LCFA respiration in brown adipocytes. (E–H) Seahorse measurements of OCR after siRNA‐mediated knockdown at day 6 of adipogenesis in immortalized brown adipocytes (imBA) with subsequent evaluation at day 9. (E) Mitochondrial FFA oxidation stress test for OA and (H) quantification of OCR for basal respiration, maximal respiration and spare respiratory capacity in siCtrl (scrambled siRNA) and siMB‐treated imBA (*n* = 6). (G) Mitochondrial FFA oxidation stress test for BSA‐conjugated octanoate (OC) and (H) quantification of OCR for basal respiration, maximal respiration and spare respiratory capacity in siCtrl and siMB treated imBA (*n* = 6). (I) Time‐resolved measurement of basal and CL stimulated uptake of palmitate and (J) area under the curve in siCtrl and siMB‐treated imBA (*n* = 5 per group). (K) Relative mRNA expression of WThMB, mutated human 4xhMB or endogenous mouse myoglobin (mMB) in fully differentiated stable overexpression imBA clones. (L) Representative capillary‐based immunoassays detecting MB protein levels in fully differentiated stable overexpression imBA clones. (M) Representative immunofluorescence staining of MB in fully differentiated stable overexpression imBA clones (scale bar: 20 µm for 400x magnification). (N) Time‐resolved measurement of basal and CL (10 nm; 100 nm) stimulated uptake of palmitate and (O) area under the curve in 4xhMB and hMB imBA clones (*n* = 5 per group). (P) Basal and CL (10 nm; 100 nm) stimulated lipolysis in 4xhMB and hMB imBA clones (*n* = 3 per group). Data are shown as mean ± SEM. Statistical significance is indicated by asterisks (^∗^ *p* < 0.05, ^∗∗^ *p* < 0.01, ^∗∗∗^ *p* < 0.001) and was determined by two‐way ANOVA with Šidák post hoc test (B, D, F, H, J, K, O, P).

Adipocytes were tested for oxidation of BSA‐conjugated oleate (OA, C18:1), a FFA with significantly higher serum concentrations in all ATMBKO mice compared to WT littermates. The specific oxidation of OA is most prominently observed in the maximal oxygen consumption rates after uncoupling of the proton gradient by FCCP. This was significantly reduced in SVF‐derived primary brown adipocytes from whole‐body MBKO mice (Figure S5A–C) compared to brown adipocytes isolated from WT littermates (Figure 5A–D) leading to reduced spare respiratory capacity in adipocytes without MB. Addition of etomoxir, blocking CPT1A and shuttling of LCFAs into mitochondria, reduced the oxygen consumption rate for both MBKO and WT adipocytes to levels of cells incubated without OA demonstrating the specific respiration of the exogenous LCFA (Figure 5A,B). Acute respiration of OA after adrenergic stimulation with norepinephrine is also significantly decreased in primary brown adipocytes from MBKO mice (Figure 5C,D).

siRNA‐mediated knockdown of MB in immortalized brown adipocytes led to a significant reduction in CL316,243 (CL)‐induced acute respiration, maximum and spare respiratory capacity (Figure S5D,E) as we have also reported previously [13]. Here, MB knockdown also caused lower basal, maximal, and spare respiratory capacity in siMB cells incubated with OA compared to control transfected cells (Figure 5E,F). Further, oxidation of BSA‐conjugated palmitate (PA, C16:0) was also significantly reduced (Figure S5G,H) and etomoxir reduced oxidation of both LCFAs. This demonstrates an impaired capacity to utilize exogenous LCFAs as substrates for mitochondrial respiration upon loss of MB. MCFAs can passively diffuse through the mitochondrial membrane and are independent of CPT1A mediated shuttling. To test if MB also promotes oxidation of MCFAs, we used BSA‐conjugated octanoate (OC, C8:0) in the FFA oxidation assay. Efficient utilization of MCFAs as substrates for mitochondrial respiration is reflected by very high basal and maximal respiration rates upon addition of OC in both control and siMB cells (Figure 5G,H). Notably, knockdown of MB also significantly reduced maximal and spare respiratory capacity fueled by OC (Figure 5G,H), and as expected, etomoxir had no effect on oxygen consumption rates in cells incubated with OC (Figure 5H).

Since we observed elevated serum lipid levels in ATMBKO mice without changes in the expression of lipid transporters, we tested if MB expression also affected adipocyte lipid uptake. Therefore, we measured basal and CL‐induced PA uptake and both were significantly lower after knockdown of MB compared to control cells (Figure 5I–K). Using our established non‐lipid binding mutant MB encoding expression vector [13], we generated imBA clones with stable overexpression of either WT human MB (hMB) or non‐lipid binding MB (4xhMB). To test whether direct binding of lipids to MB is responsible for the increased lipid flux, we selected clones with matched protein overexpression of WT and mutant MB (Figure 5L,M). As previously reported, clones expressing the non‐lipid binding 4xhMB respond to adrenergic activation with lower PKA activity and have lower mitochondrial respiratory capacity (Figure S5I–K). Additionally, lipid uptake (Figure 5N,O) and lipolysis rates (Figure 5P) are significantly lower in 4xhMB clones compared to adipocytes expressing WT hMB. Together, these findings indicate that MB plays a central role in multiple aspects of lipid handling, including lipid uptake, lipolysis, and mitochondrial beta‐oxidation, by acting as a lipid‐binding protein that regulates intracellular fatty acid availability and thereby coordinates the metabolic pathways required for thermogenesis.

### 2.6 Adipose MB Expression is Upregulated by HFD Feeding and Under the Control of Thyroid Hormones

**FIGURE 6:**
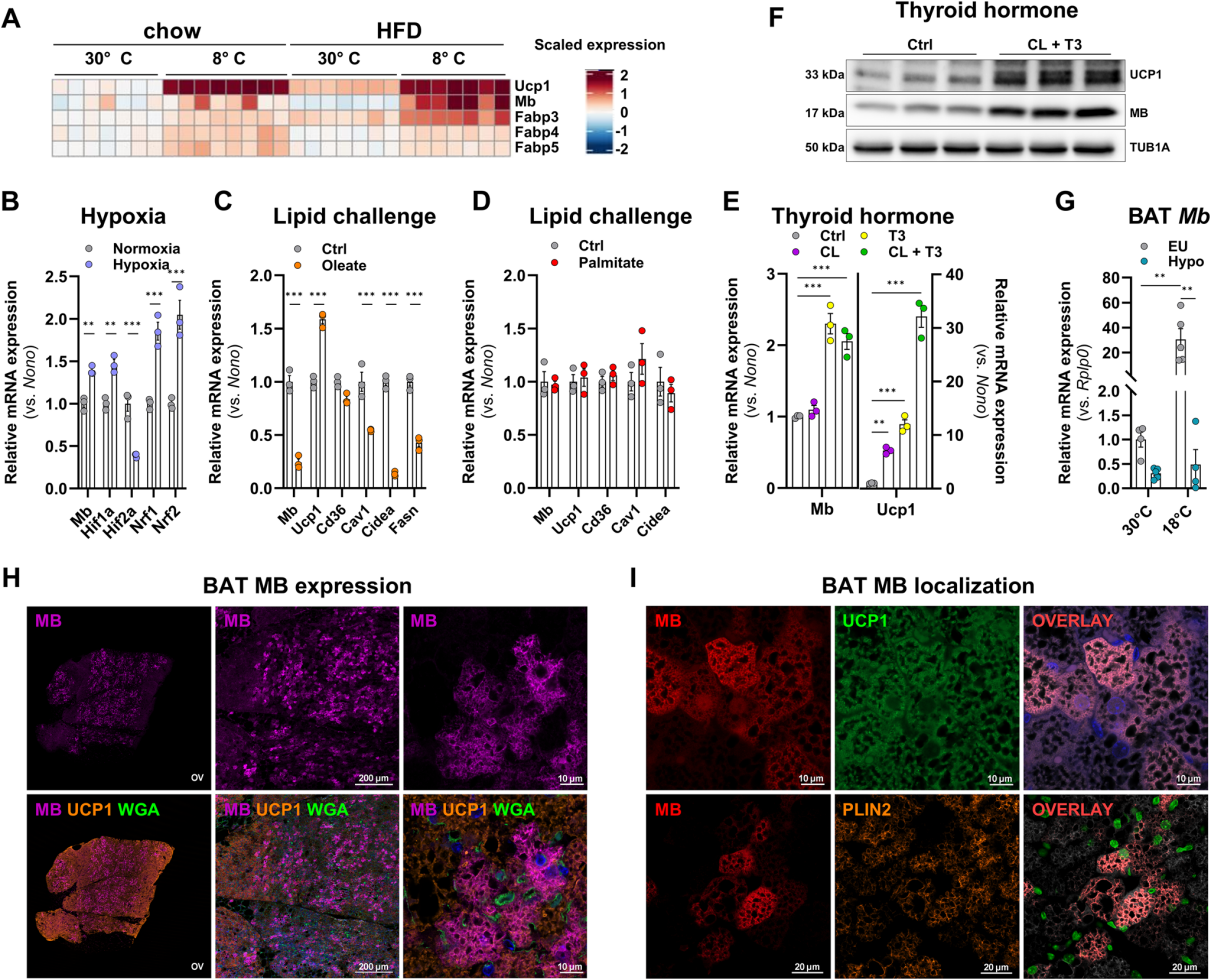
Regulatory mechanisms of MB expression in BAT. (A) Scaled expression of Ucp1, Mb, Fabp3, Fabp4, and Fabp5 in BAT of mice fed either a chow or a high fat diet (HFD) and housed at either thermoneutrality or in the cold for one week. (B–E) Effects of hypoxia, lipid challenge, and thyroid hormones on Mb expression in immortalized brown adipocytes (imBA). (B) Relative mRNA expression of Mb and hypoxia responsive Hif1a, Hif2a, Nrf1 and Nrf2 in imBA cells cultivated at 2% O_2_ for 16 h compared to cells cultivated at normoxia. (C‐D) Relative mRNA expression of Mb, Ucp1 and lipid handling (Cd36, Cav1, Cidea, Fasn) genes in imBA cells stimulated with (C) BSA‐conjugated oleate (OA; 100 µm) or (D) BSA‐conjugated palmitate (PA; 100 µm) for 24 h compared to unstimulated control cells (*n* = 3 per condition). (E) Relative mRNA expression of Mb and Ucp1 in imBAs stimulated with either CL (100 nm), Triiodothyronine (T3; 10 nm), or CL and T3 (100 nm; 10 nm) for 24 h compared to unstimulated control cells (*n* = 3 per condition). (F) Representative Western blot analysis of MB and UCP1 in imBAs stimulated with CL and T3 (100 nm; 10 nm) for 24 h and in unstimulated control cells (*n* = 3 per condition). Alpha‐tubulin served as a loading control. (G) Mb and Ucp1 expression in BAT of WT eu‐ and hypothyroid (EU and Hypo respectively) mice housed at thermoneutrality (30°C) or exposed to cold (18°C) for 7 days (*n* = 4 for EU 30°C and Hypo 18°C and *n* = 5 for Hypo 30°C and EU 18°C). (H) Immunofluorescence staining of BAT from HFD‐fed WT mice after 5 days of cold exposure. UCP1, MB, and wheat germ agglutinin (WGA) co‐staining depicting representative MB expression, respectively (scale bar: 200 µm for 50x magnification, 10 µm for 630x magnification). (I) Immunofluorescence staining of BAT from HFD‐fed WT mice after 5 days of cold exposure. Representative MB co‐staining with UCP1 and PLIN2 with an overlay of MB signal with UCP1 and PLIN2, respectively (scale bar: 20 µm for 400x magnification, 10 µm for 630x magnification). Data are shown as mean ± SEM. Statistical significance is indicated by asterisks (^∗^ *p* < 0.05, ^∗∗^ *p* < 0.01, ^∗∗∗^ *p* < 0.001) and was determined by two‐way ANOVA with Šidák post hoc test (C, D, H) and one‐way ANOVA with Šidák‐Holm post hoc test (F; separately for Mb and Ucp1).

Diets rich in fat lead to a systemic increase in circulating lipids, increasing the need for lipid mobilization and oxidation in BAT. We analyzed MB expression in comparison to other fatty acid binding proteins (FABP) in BAT of mice that were either fed a chow or an HFD and housed at thermoneutrality or in the cold for one week [53]. As previously shown, cold acclimation induced MB expression in BAT. Intriguingly, HFD feeding and cold exposure together induced the most pronounced increase in MB expression, by far the most dramatic change or response among the fatty acid binding proteins (Figure 6A). While FABP3 (heart‐type FABP) showed a similar regulatory pattern, FABP4 (adipocyte FABP) and FABP5 (epidermal FABP) were not cold‐responsive at all. Higher MB expression in HFD‐ compared to chow‐fed mice after cold exposure is confirmed in the WT littermates of ATMBKO mice used in this study (Figure S5L).

In our previous work [13], we systematically investigated MB expression in vitro using established thermogenic and adrenergic stimuli, including norepinephrine, CL, rosiglitazone, and menthol. These treatments did not induce MB expression in cultured adipocytes, suggesting that MB is not directly regulated by canonical adrenergic signaling in vitro. To explore possible drivers of the observed induction of MB expression under a HFD, we tested additional regulatory cues. These include hypoxia (Figure 6B), hyperlipidemic conditions using oleate or palmitate (Figure 6C,D), and triiodothyronine (T3) (Figure 6E). Among these, T3 robustly induced MB expression in adipocytes both on mRNA and protein level (Figure 6F and Figure S5M). Further, analysis of mRNA transcript levels of MB in euthyroid and hypothyroid animals housed at thermoneutrality or mild cold (18°C) revealed that cold exposure failed to induce MB expression in hypothyroid mice, in contrast to the physiologic response observed in euthyroid mice (Figure 6G). Interestingly, the oleate challenge reduced *Mb* expression together with genes responsible for lipid uptake and handling while palmitate had no effect and hypoxia slightly increased *Mb* gene expression in vitro.

Immunofluorescence co‐staining of MB and UCP1 shows the expression pattern of MB in BAT of cold acclimated and HFD fed WT mice (Figure 6H). We further investigated the cellular localization of MB assessing co‐localization of MB with UCP1 and PLIN2. UCP1 localizes in the inner mitochondrial membrane of mitochondria [54, 55], and MB has been detected within the inner mitochondrial membrane in both muscle cells [56] and brown adipocytes [13]. Overlay of UCP1 and MB signals confirmed co‐localization of both proteins in brown adipocytes of WT mice exposed to cold (Figure 6I). Furthermore, we observed prominent MB staining surrounding intracellular lipid droplets (Figure S5N), prompting us to perform co‐staining of MB and PLIN2 (perilipin‐2), a protein that binds directly to lipid droplets. Indeed, we found co‐localization of both proteins, suggesting that MB is also present in the immediate vicinity of lipid droplets (Figure 6I).

### 2.7 ATMBKO Mice Exhibit Preserved Mitochondrial Integrity and Function

**FIGURE 7:**
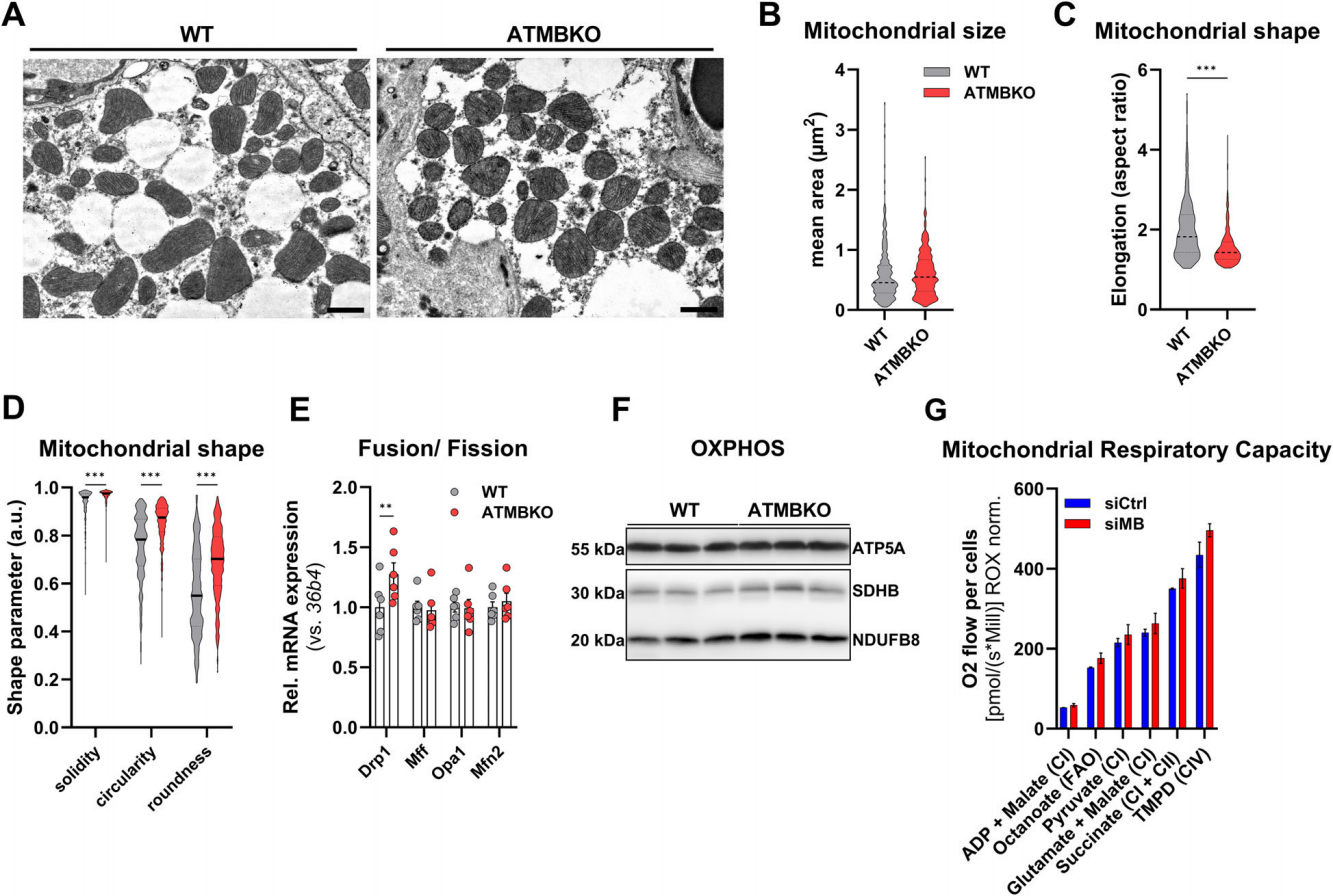
Mitochondria in BAT of ATMBKO mice are more fragmented but remain intact. (A) Representative electron microscopy image of mitochondria from BAT of HFD‐fed ATMBKO and WT mice after 5 days of cold exposure, with measured mitochondrial (B) size, (C) elongation, and (D) shape parameters. (E) Relative mRNA expression of fusion/fission marker genes (*n* = 6) in BAT of HFD‐fed ATMBKO and WT mice after 5 days of cold exposure. Gene expression is normalized to 36b4. (F) Western blot analysis for OxPhos protein complexes in BAT of HFD‐fed ATMBKO and WT littermates after 5 days of cold exposure. (G) Oroboros measurements of mitochondrial respiratory capacity in permeabilized imBAs after siRNA‐mediated knockdown of Mb (*n* = 2 parallel measurements in different O2k respirometers). Data are shown as mean ± SEM. Statistical significance is indicated by asterisks (^∗^ *p* < 0.05, ^∗∗^ *p* < 0.01, ^∗∗∗^ *p* < 0.001) and was determined by Student's *t*‐test (C) and two‐way ANOVA with Šidák post hoc test (D, E).

Finally, we analyzed mitochondrial morphology and integrity in BAT by electron microscopy, comparing HFD‐fed and cold‐exposed ATMBKO and WT littermates. Mitochondria from UCP1‐KO mice were shown to display severe alterations beyond the absence of UCP1, including loss of electron transport chain protein abundance [57]. This was not observed in ATMBKO mice, however morphologically, mitochondria from ATMBKO mice displayed increased circularity, roundness, solidity, and elongation compared to WT littermates, but no differences in overall mean size (Figure 7A–D). It is well established that mitochondria dynamically respond via fusion and fission to different metabolic demands as well as stress signals, thereby altering the mitochondrial network morphology [58]. Increased mitochondrial circularity, roundness, and solidity suggest a shift towards mitochondrial fission in BAT of ATMBKO mice. Gene expression analysis of key mediators of fusion and fission dynamics confirmed increased expression for *Drp1* (dynamin‐related protein) (Figure 7E), the main mediator of mitochondrial fission, in ATMBKO mice. Abundance of oxidative phosphorylation complexes *I*–*V* was unchanged between ATMBKO and WT animals (Figure 7F) indicating no difference in oxidative capacity. To further test mitochondrial integrity, we analyzed mitochondrial respiratory capacity in permeabilized brown adipocytes after knockdown of MB. Mitochondrial respiration of permeabilized cells is independent of intracellular shuttling systems as mitochondria have direct contact with substrates in the medium. Mitochondrial respiratory capacity assessed by substrate titration was unchanged in siMB‐treated cells compared to control cells (Figure 7G and Figure S5N,O).

### 2.8 MB Enhances Lipolysis and Mitochondrial Respiration in Human Adipocytes

**FIGURE 8:**
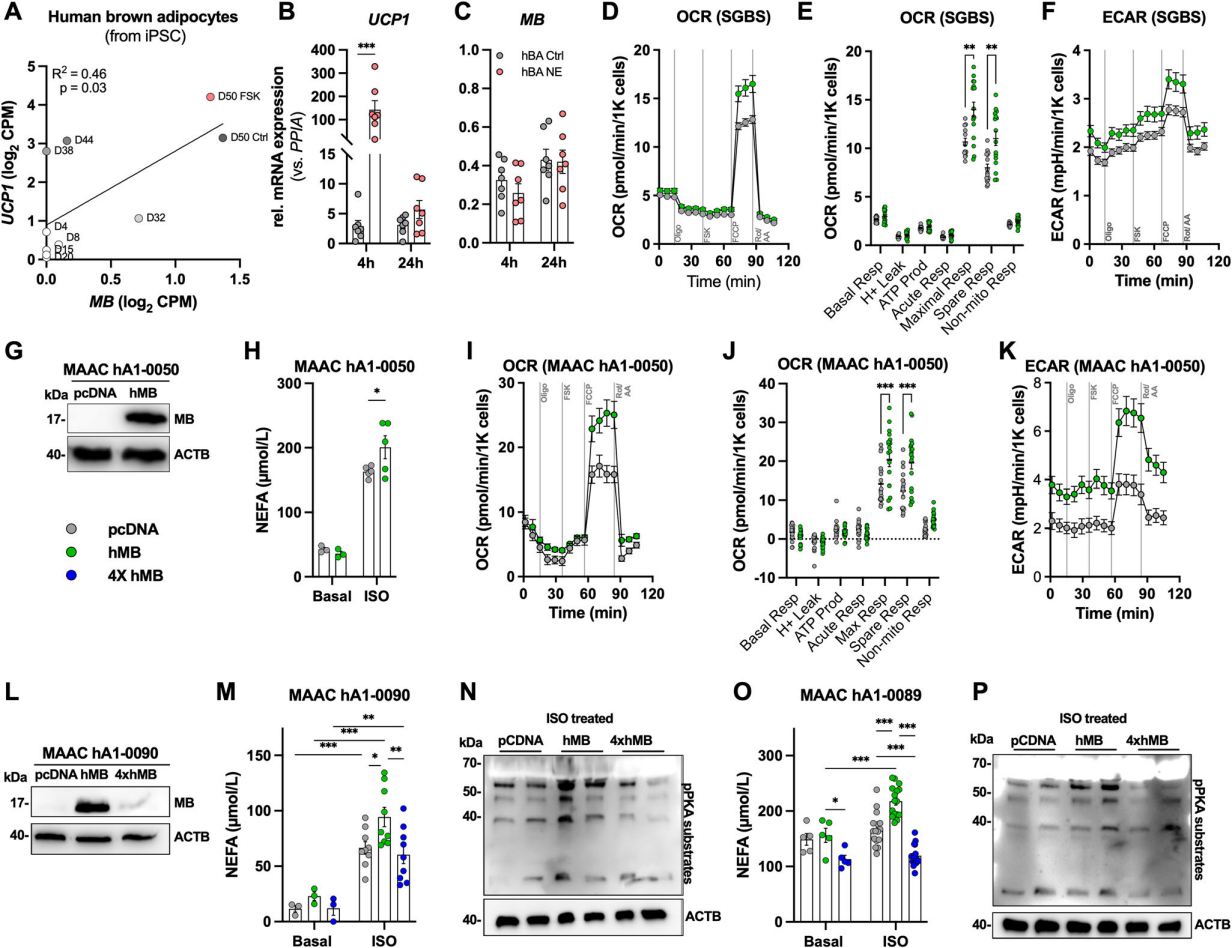
MB is expressed in human brown adipocytes and promotes oxidative metabolism. (A) Publicly available RNA‐sequencing data from human pluripotent stem cell‐derived brown adipocytes [106] show detectable MB mRNA expression emerging in late stage of differentiation (day 44–50). MB expression correlates with UCP1 expression (*n* = 1 for day 4–44, *n* = 3 for day 50 Ctrl/ FSK, R^2^ = 0.43, *p* = 0.03). (B) UCP1 and (C) MB expression in human brown adipocytes differentiated from primary adipose stromal vascular fraction (SVF) cells and exposed to norepinephrine (NE) for 4 and 24 h (*n* = 7). (D‐F) Mitochondrial Stress Test with acute injection of forskolin (FSK) in hMB or pcDNA transfected SGBS cell. (D) OCR profile and (E) quantification of basal respiration, proton leak, ATP production, acute respiration, maximal respiration, spare respiratory capacity, and non‐mitochondrial respiration, and (F) extracellular acidification rate (ECAR) profile (*n* = 15 replicates, experiment conducted twice). (G–L) Overexpression of MB (hMB) in primary human mature adipocytes isolated from one donor (hA1‐0050) and cultured as membrane mature adipocyte aggregate cultures (MAAC). (G) Representative Western blot analysis confirming increased MB protein levels after electroporation of hMB plasmid in membrane mature adipocytes. (H) Basal and isoproterenol‐ (ISO) stimulated lipolysis in mature adipocytes (*n* = 3 (Ctrl)/5 (ISO)). (I–K) Mitochondrial Stress Test with acute injection of forskolin (FSK) in hMB or pcDNA transfected mature adipocytes. (I) OCR profile and (J) quantification of basal respiration, proton leak, ATP production, acute respiration, maximal respiration, spare respiratory capacity, and non‐mitochondrial respiration. (K) ECAR profile (*n* = 19 replicates per condition). (L–P) Overexpression of hMB and non‐lipid binding MB (4xhMB) in primary human mature adipocytes (hA1‐0090 and hA1‐0089). (L) Representative Western blots showing MB protein expression after electroporation of hMB and 4xhMB plasmid. (M) Basal and isoproterenol (ISO)‐stimulated lipolysis (*n* = 3 (basal)/9 (ISO)) and (N) Western blot analysis of phosphorylated PKA substrates after ISO stimulation (100 nm, 60 min) in transfected human adipocytes (hMB, 4xhMB or pcDNA plasmid); beta‐actin (ACTB) served as a loading control (*n* = 2 per group; hA1‐0090). (O) Basal and ISO‐stimulated lipolysis (*n* = 5 (basal)/16 (ISO)) and (P) Western blot analysis of phosphorylated PKA substrates after ISO stimulation (100 nm, 60 min) in transfected human adipocytes (hMB, 4xhMB or pcDNA plasmid); ACTB served as a loading control (*n* = 2 per group; hA1‐0089). Data are presented as mean ± SEM. Statistical significance is indicated by asterisks (^∗^ *p* < 0.05, ^∗∗^ *p* < 0.01, ^∗∗∗^ *p* < 0.001) and was determined by simple linear regression (A) or two‐way ANOVA with Šidák post hoc test (B, E, K, N, P) or with Fisher's LSD test (H,I).

To determine whether the regulatory role of MB in lipid handling and oxidative metabolism is conserved in humans, we investigated MB expression and function in human adipocytes using primary human adipocyte models and SGBS cells. Analysis of a publicly available RNA‐seq dataset of human brown adipocytes differentiated from iPSCs revealed detectable MB expression throughout adipogenic differentiation, with a pronounced increase at late differentiation stages (Figure 8A). MB expression at day 50 (mature adipocytes) was substantially elevated compared with earlier time points (day 4–44) and correlated with UCP1 expression. Next, we validated MB expression in primary human brown adipocytes derived from supraclavicular AT. MB mRNA was consistently detected under basal conditions and remained unchanged following 4 and 24 h norepinephrine stimulation, while UCP1 mRNA was induced after 4 h of NE (Figure 8B,C). These findings mirror our in vitro observations in isolated murine adipocytes where adrenergic stimulation alone failed to induce MB expression [13].

To test if MB expression would increase oxidative metabolism of human adipocytes, we first overexpressed human MB in SGBS cells (Figure S6A). We performed seahorse mitochondrial stress test and overexpression of MB enhanced mitochondrial respiratory activity, resulting in significantly higher maximal respiration and spare respiratory capacity compared with controls (Figure 8D,E). Also, ECAR was elevated in SGBS cells overexpressing hMB. Next, using human mature adipocytes cultured as membrane aggregate adipocyte cell culture (MAAC), we induced overexpression of human MB in primary white adipocytes. Immunoblot analysis confirmed successful overexpression of human MB in mature adipocyte (Figure 8G). Functionally, MB overexpression significantly increased ISO‐stimulated lipolysis, reflected by an elevated release of non‐esterified fatty acids (NEFAs) mature adipocytes compared with control transfected cells (Figure 8H). Seahorse mitochondrial respiration analysis in MAAC demonstrated that hMB overexpression also enhanced mitochondrial oxidative capacity in primary human adipocytes. Maximal respiration and spare respiratory capacity were significantly higher compared with controls (Figure 8I,J and Figure S6B,C) and consistent with SGBS cells. In agreement with an increased energetic demand, ECAR was also elevated suggesting enhanced glycolytic support for mitochondrial metabolism (Figure 8K and Figure S6D).

To confirm that the metabolic effects of MB in human adipocytes depend on its lipid‐binding capacity, we transfected primary human MAAC adipocytes with either hMB or non‐lipid‐binding 4xhMB. Immunoblot analysis confirmed expression of both constructs, although the 4xhMB mutant consistently showed a weaker signal, as previously reported (Figure 8L). Functionally, overexpression of MB significantly increased ISO‐stimulated lipolysis, as reflected by enhanced NEFA release and increased phosphorylation of PKA substrates in adipocytes from two independent donors (Figure 8M–P). In contrast, adipocytes expressing the non‐lipid‐binding 4xhMB mutant displayed lipolytic responses comparable to control cells, and in one donor even showed reduced lipolysis. Notably, MB overexpression was sufficient to enhance lipolysis even in adipocytes from a donor with low responsiveness to ISO stimulation, indicating that MB can augment adrenergic lipolytic capacity in otherwise resistant human adipocytes (Figure 8O,P).

Together, these results demonstrate that MB is expressed in human brown adipocytes and that it is of functional relevance in human adipocytes by enhancing lipid mobilization and oxidative metabolism.

### 2.9 MB Re‐Expression in MBKO Mice Limits HFD‐Induced Weight Gain

**FIGURE 9:**
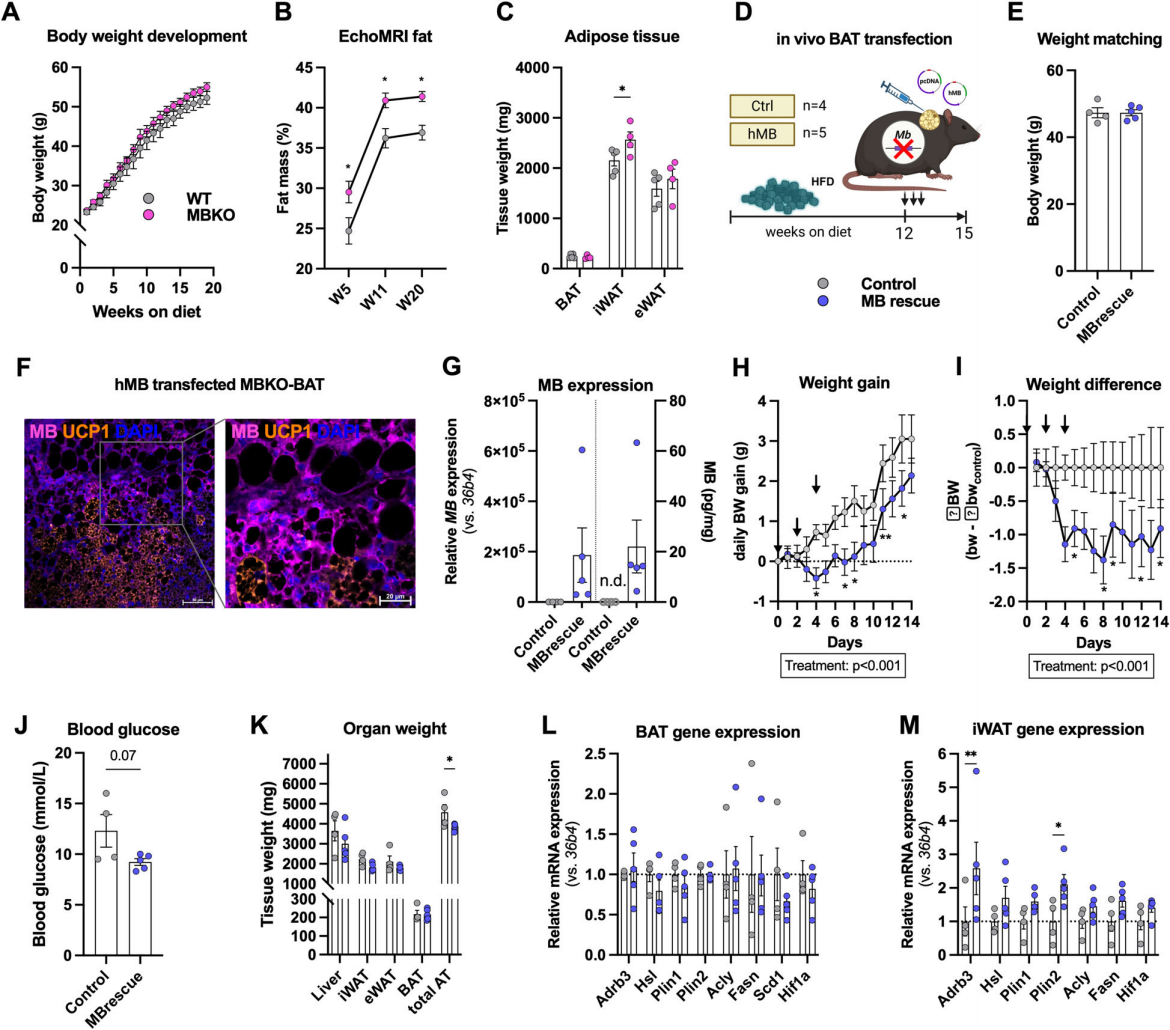
Rescue of MB expression in MBKO mouse BAT limits diet‐induced weight gain and improves metabolic health. (A) Body weight development of male HFD‐fed WT and MBKO mice from 5 to 24 weeks of age. Diet started at 5 weeks of age (*n* = 5/4). (B) Percentage of fat mass determined by EchoMRI after 5, 11, and 20 weeks on diet in male WT and MBKO mice (*n* = 5/4). (C) AT weights after 20 weeks on a HFD in male MBKO and WT mice (*n* = 5/4). (D) Schematic depicting experimental design for re‐expression of MB in HFD‐fed MBKO mice by in vivo‐jetPEI mediated plasmid transfection. Created in BioRender, https://BioRender.com/5ts4hlf. (E) Body weight matched mice for MB‐transfection (MB rescue) and control groups after 12 weeks on the HFD (*n* = 4/5, for all following panels). (F) Representative immunofluorescence co‐staining of MB and UCP1 in BAT from mice transfected with hMB three days prior to staining of the tissue (scale bar: 50 µm for 200x magnification, 20 µm for 400x magnification). (G) Relative mRNA and protein expression of MB in BAT of MB rescue and control mice at the end of the study (21 days after first injection, 20 weeks of age). (H) Daily body weight gain and (I) relative body weight difference of MB rescue mice compared to controls during the first 14 days of the study. (J) Blood glucose concentration at the end of the study. (K) Organ weights and (L,M) mRNA expression of genes involved in thermogenic activation and lipid handling in (L) BAT and (M) iWAT of MB rescue and control mice at the end of the study. Gene expression is normalized to *36b4* and relative to control mice. Protein expression was analyzed by ELISA. Data are shown as mean ± SEM. Statistical significance is indicated by asterisks (^∗^ *p* < 0.05, ^∗∗^ *p* < 0.01, ^∗∗∗^ *p* < 0.001) and was determined by two‐way ANOVA with Šidák post hoc test (B) or Fisher's LSD test (C, H, I, K, M) and Student's t‐test (J).

Finally, we chose the whole‐body MBKO mouse model to perform a proof‐of‐concept study rescuing MB expression in BAT. We observed that MBKO mice displayed increased susceptibility to diet‐induced obesity with higher body weight and fat mass compared to WT littermate animals (Figure 9A–C; in trend also previously observed [59]). To assess whether restoration of MB expression in BAT can improve the metabolic phenotype of whole body MBKO mice, we performed an in vivo rescue experiment using transient plasmid‐based gene delivery. MBKO mice fed a high‐fat diet (HFD) for twelve weeks where randomized for body weight and received repeated local injections of a human MB expression plasmid or control plasmid directly above the interscapular BAT at 2‐day intervals using the established in vivo‐jetPEI transfection reagent (Figure 9D,E). Analysis of MB expression in control animals two days post injection confirmed high level MB protein expression (Figure 9F), that was still detectable at the end of the MB‐rescue experiment (Figure 9G, mRNA and protein expression). Remarkably, re‐introduction of MB in BAT resulted in a transient but significant reduction in body weight gain compared to control transfected MBKO mice (Figure 9H,I). This effect was apparent 24 h after the first injection, indicating rapid functional consequences of MB expression in BAT. Body weight changes remained lower in MB transfected mice for 9 days until 72 h after the final injection. This pattern is consistent with the transient nature of plasmid‐based gene expression. MB‐rescue mice also showed lower blood glucose levels (*p* = 0.07) and overall significantly lower AT mass (Figure 9J,K). Except for MB, we did not find significant changes in thermogenic or lipid metabolism gene expression in transfected BATs (Figure 9L). However, significant increases in iWAT *Adrb3* and *Plin2*, together with overall trends for higher *Hsl*, *Plin1*, *Acly* and *Fasn* suggest metabolic adaptation of iWAT that may fuel increased BAT thermogenesis (Figure 9M).

Notably, these effects were achieved despite the weak and transient nature of plasmid‐based expression, suggesting that more efficient and sustained approaches such as mRNA delivery or AAV‐mediated gene transfer could substantially enhance the therapeutic impact of MB restoration. Together, these findings demonstrate that restoring MB specifically in BAT is sufficient to improve metabolic outcomes in MB‐deficient mice, highlighting its functional role in regulating energy balance.

## 3 Discussion

### 3.1 Knockout of MB in AT Leads to Impaired Thermoregulation and Reduced Energy Expenditure in Cold

Previous studies have demonstrated the expression of MB in BAT [14, 15] and suggested a role in BAT lipid metabolism [16, 17], before subsequently demonstrating that MB drives adrenergic activation, lipolysis, and mitochondrial respiration of brown adipocytes via its lipid‐binding properties [13]. On the cellular level, recent BAT single nuclei sequencing revealed MB to be specifically expressed in myogenic or contractile brown adipocytes, a unique cluster of high level‐UCP1 expressing brown adipocytes [41]. Furthermore, also white adipocytes residing in or around the brown fat depot express low levels of MB [60], and it is very abundant in UCP1‐positive beige adipocytes within the iWAT of cold‐exposed mice. Consistent with previous findings of increased iWAT MB expression after pharmacologic BAT activation using CL and enhanced metabolic activity in both brown and white adipocytes overexpressing MB [13], this suggests an important functional role for MB in thermogenic adipocytes in general, both beige and brown.

Unexpectedly, chow‐fed male ATMBKO mice had lower body weights compared to littermate controls, but ATMBKO mice had a significantly higher body fat content in general, specifically more iWAT and BAT mass. This suggests a dysbalance in lipid metabolism in ATMBKO, resulting in increased fat accumulation offset by an even more pronounced reduction in lean mass due to elevated protein catabolism. Indeed, serum metabolomics showed accumulation of lipids and a reduction of AAs and AA‐related metabolites in ATMBKO mice. Elevated levels of plasma lipids result in dyslipidemia and are hallmarks of obesity and promote the development of type 2 diabetes [61], impairing beta cell function and insulin secretion in response to glucose without already affecting peripheral insulin sensitivity. Consistently, ATMBKO mice exhibited significantly worse glucose clearance rates albeit no changes in insulin sensitivity.

During prolonged cold exposure, ATMBKO mice exhibited reduced energy expenditure and impaired thermoregulation. Underlying were transcriptional changes in BAT of cold‐exposed ATMBKO mice, with significant downregulation of pathways involved in oxidative phosphorylation, adipogenesis, myogenesis, and fatty acid metabolism. Conversely, the transcriptome was largely unchanged between ATMBKO and WT mice at thermoneutrality, when BAT is inactive. These results demonstrate the importance of MB expression for the metabolic activity and function of thermogenic adipocytes in vivo. Contractile processes in brown adipocytes contribute to BAT activation and thermogenesis and two recent studies described a contractile brown adipocyte subpopulation with enriched expression of muscle genes [44, 62]. We find that MB is a marker for the contractile brown adipocyte subpopulation in both data sets, and that loss of MB leads to downregulation of the contractile marker genes, indicating a loss of the contractile phenotype and demonstrating the substantial contribution of this adipocyte subcluster to energy expenditure. Importantly, the ATMBKO model allows for the exclusion of muscle‐related alterations that contribute to energy expenditure, which were previously difficult to differentiate using whole‐body MBKO mice [13].

### 3.2 Reduced Energy Expenditure Increases Susceptibility to Diet‐Induced Obesity in HFD‐Fed ATMBKO Mice

A chronic energy surplus induced by high caloric diets is the most common environmental factor leading to obesity and metabolic disease. In this context, MB expression in BAT is dynamically regulated by thermogenic activity and closely interacts with nutritional status [53]. Consistent with its role in BAT lipid handling, induction of MB is particularly pronounced in cold‐activated BAT of HFD‐fed mice where substrate availability and thermogenic demand are both elevated. However, hyperlipidemic conditions alone were not sufficient to induce MB expression in vitro, indicating that nutrient excess per se does not directly regulate MB. Instead, thyroid hormone signaling emerged as a critical upstream regulator, as it robustly induced MB expression and was required for cold‐induced MB upregulation in vivo. These findings suggest that MB expression is controlled by the integration of thermogenic and endocrine signals rather than by lipid availability alone, positioning MB as part of a coordinated adaptive program linking hormonal activation with metabolic substrate utilization.

Under an HFD, knockout of adipose MB caused an additional weight gain of 10% compared to WT littermates after 20 weeks on the diet, in both male and female mice. ATMBKO mice had significantly more body fat and increased adipocyte hypertrophy in iWAT, as well as aggravated glucose intolerance. More pronounced obesity was driven by significantly lower whole‐body energy expenditure especially during the active phase, independent of food intake, locomotor activity, and ambient temperature (i.e. even at thermoneutrality). BAT metabolic activity and substrate uptake peak around the onset of the active phase [63, 64] demonstrating the importance of MB for diet‐induced and lipid fueled thermogenesis. After food intake and under high caloric diets, BAT is activated to act as a catabolic sink and clear macronutrients from the circulation, thereby protecting the body from nutritional overflow [65, 66]. Studies in humans have shown that higher glucose and lipid metabolism is associated with active BAT based on ^18^F‐FDG PET/CT imaging at room temperature (i.e., human thermoneutrality) [4, 7, 8, 67, 68]. Therefore, MB expression in AT seems to be an important factor in counteracting diet‐induced obesity by enabling BAT to burn excess dietary lipids. Consistently, thermogenesis, body temperature, and energy expenditure in response to cold were impacted more strongly in HFD‐fed ATMBKO mice.

### 3.3 MB Knockout in AT Changes Lipid and BCAA Metabolome

Loss of MB in AT leads to pronounced and widespread changes in the serum metabolome of mice exposed to cold or fasting. Cold and fasting trigger SNS activity, which leads to WAT lipolysis. FFAs can then be used directly as fuel for BAT thermogenesis or indirectly routed through the liver, where they are synthesized to acylcarnitines or re‐esterified into TGs and packaged into TG‐rich lipoproteins. In rodents, BAT activity is the main driver of TG clearance from the blood in response to short‐term cold exposure [9]. Targeted metabolomics confirmed that short‐term (24 h) cold exposure leads to drastic reductions of serum TGs in male WT mice compared to littermates housed at thermoneutrality. Compared to WT mice, serum TGs as well as DGs are highly elevated in male ATMBKO mice after cold exposure, and we observed the same in female HFD‐fed ATMBKO mice challenged by an overnight fast. In brown adipocytes, knockdown and knockout of MB limited fatty acid uptake and mitochondrial oxidation of exogenous long‐ and medium‐chain fatty acids. In vivo, and consistent with impaired lipid mobilization, uptake and utilization, fasted ATMBKO mice exhibited a significant reduction in body temperature compared to WT littermates.

Serum concentrations of AAs and especially BCAAs are significantly lower in all ATMBKO mice, after cold exposure and fasting. Recent studies suggest that BAT utilizes circulating BCAA as fuel for thermogenesis [9, 69] and that BAT is responsible for ∼20% of whole‐body BCAA oxidation at room temperature, making it the organ with the second highest BCAA oxidation after skeletal muscle [70]. Moreover, skeletal muscle can supply AA as fuel to support BAT thermogenesis. Proteolysis in skeletal muscle liberates AA, particularly BCAAs, which are subsequently oxidized by BAT during cold exposure [71]. There, muscle‐derived IL6 stimulates BCAA release from skeletal muscle while simultaneously promoting BAT thermogenic activity, thereby establishing a compensatory muscle‐BAT axis [71]. In BAT, the mitochondrial BCAA transporter SLC25A44 is critical for BCAA catabolism and gene knockout impairs thermogenesis. *Slc25a44*, along with the BCAA catabolic pathway enzymes *Bcat2* and *Bcdha/ b*, is upregulated in BAT of cold‐acclimated ATMBKO mice. Together with the significantly reduced serum concentrations of BCAAs, this suggests a compensatory increase in BCAA catabolism in BAT of ATMBKO mice. Additionally, the IL6‐JAK‐STAT signaling pathway is enriched in BAT of chow‐fed and cold exposed ATMBKO mice. Therefore, increased AA oxidation together with limited lipid utilization could account for the reduction of body weight with reduced lean and increased fat mass observed in male chow‐fed ATMBKO mice. Future studies will address whether lack of AT expressed MB may aggravate loss of lean or muscle mass during weight loss induced by bariatric surgery or incretin‐mimetic drugs [72].

Furthermore, the level of mTOR phosphorylation at Ser2481 was significantly higher in BAT from cold‐adapted ATMBKO mice, indicating elevated mTORC1 activity. BCAAs, especially leucine, are potent activators of mTORC1 [73]. mTORC1 regulates cell growth and metabolism [74] and in BAT, deletion of mTORC1 induces severe alterations in lipid metabolism in response to cold [75]. mTORC1 is a central regulator of anabolic metabolism, and its activation promotes de novo lipogenesis primarily through the transcriptional control of SREBP1c. Consistently, we observed an upregulation of lipogenesis genes in BAT and iWAT of ATMBKO mice that was independent of diet and has also been reported in whole‐body MBKO mice [16]. Enhanced mTORC1 signaling may be an adaptive, compensatory response that redirects carbon flux toward de novo lipogenesis when lipid uptake and oxidation are impaired in ATMBKO.

### 3.4 MB Expression Affects Mitochondrial Fusion and Fission

Upon cold stimulation, brown adipocytes undergo extensive mitochondrial network fragmentation that promotes increased uncoupled respiration and fatty acid oxidation [76]. This mitochondrial fragmentation is evident in BAT of HFD‐fed and cold‐exposed WT and ATMBKO mice. However, WT BAT displayed a larger number of elongated mitochondria, while we found increased mitochondrial fission in BAT of ATMBKO. Importantly, analyses in permeabilized cells showed no changes in mitochondrial integrity nor respiratory capacity. Mitochondrial dynamics modulate the interaction with intracellular organelles such as lipid droplets and these interactions impact thermogenesis. Two types of mitochondria have been identified in brown adipocytes, i.e., cytosolic and peri‐droplet mitochondria. Peri‐droplet mitochondria form tight contacts with lipid droplets and have an elongated morphology. They were shown to increase beta‐oxidation under metabolic stress conditions [77, 78], though more recent studies suggest that peri‐droplet mitochondria play a role in lipid droplet expansion [79]. MB is located in the inner mitochondrial matrix of BAT mitochondria but also found in the vicinity of lipid droplets. This indicates that MB may influence the interaction of mitochondria and lipid droplets. Further studies are needed to determine if MB influences mitochondrial dynamics in brown adipocytes directly or whether the observed shift in mitochondrial fusion and fission dynamics merely reflects changes in metabolic flexibility.

### 3.5 MB Overexpression Enhances Lipid Mobilization and Oxidative Metabolism in Primary Human Adipocytes

We have previously reported that expression of MB is higher in human AT samples that exhibit a higher thermogenic potential and is differentially expressed in human subcutaneous and visceral AT depots, where it is regulated by the state of obesity [13]. Here, we confirm that MB is expressed in primary human brown adipocytes and is functionally relevant in human adipocytes. Overexpression of MB in primary human white adipocytes significantly enhanced lipid mobilization und oxidative metabolism, demonstrating a conserved function of MB in AT metabolism. Augmenting MB expression in AT may therefore promote lipid utilization and increase energy expenditure, thereby mitigating diet‐induced weight gain and improving systemic metabolic health. Although empirical evidence is currently lacking, it is conceivable that higher adipose MB levels could indirectly attenuate the loss of muscle mass typically observed during weight loss. Modulating adipose MB expression may thus represent a complementary strategy for future obesity treatment strategies.

### 3.6 Rescue of MB Expression Improves Whole Body Metabolism In Vivo

Re‐expression of MB in BAT of MBKO mice using transient in vivo gene delivery was sufficient to acutely reduce body weight gain. The rapid onset and reversibility of this effect suggest that MB acts as a dynamic regulator of thermogenic metabolism. These findings demonstrate that restoration of MB in BAT is sufficient to acutely improve whole‐body metabolic outcomes in MBKO mice supporting a causal role for adipocyte MB in regulating energy expenditure and body weight homeostasis. But in comparison to ATMBKO mice, additional loss of MB in muscle does not seem to further increase the susceptibility to diet‐induced obesity. From a translational perspective, this suggests that increasing MB expression in AT may represent a novel strategy to enhance energy expenditure. This concept is particularly attractive in the context of current anti‐obesity therapies, which primarily act by reducing caloric intake, such as GLP‐1 receptor agonists or dual/triple incretin‐based therapies. In contrast, targeting MB could complement these approaches by increasing energy expenditure.

Future therapeutic strategies could involve transient upregulation of MB in AT, for example via mRNA‐based delivery systems with adipocyte‐specific targeting. Such approaches may enable controlled enhancement of thermogenic metabolism without requiring permanent genetic modification.

Together, our findings position adipocyte MB as a key regulator of metabolic flexibility and a potential target for interventions aimed at increasing energy expenditure and combating obesity.

## 4 Methods

### 4.1 Ethics Approval and Patient Consent

Animal studies: All animal experiments were approved by the local authorities of the State of Saxony (Regierungspräsidium Leipzig, Germany: TVV 07/22, TVV51/20, T09/21), as recommended by the responsible local animal ethics review board.

Human samples of the Leipzig Obesity BioBank (LOBB) and University of Edinburgh: All studies were approved by the Ethics Committee of the University of Leipzig (approval numbers: 159‐12‐21052012 and 017‐12ek) or University of Edinburgh (approval number: 20/ES/0061) and performed in accordance with the Declaration of Helsinki, the Bioethics Convention (Oviedo), and EU Directive on Clinical Trials (Directive 2001/20/EC). All AT donors have been informed of the purpose, risks and benefits of the biobank, and written informed consent was obtained from all patients. Ethical guidelines and EU legislation for privacy and confidentiality in personal data collection and processing is being followed, in particular directive 95/46/EC.

### 4.2 Animal Experiments

AT‐specific knockout of the MB gene was achieved by cross breeding floxed Mb^fl/fl^ mice on the C57BL/6N background (generated by Cyagen, Santa Clara, CA, USA) with mice expressing the Cre‐recombinase under the transcriptional control of the adiponectin promoter (Jackson Laboratories, B6.FVB‐Tg(Adipoq‐cre)1Evdr/J, stock no. 028020) to obtain ATMBKO mice and WT littermates. Recombination results in ubiquitous deletion of exon two with loss of function for the Mb gene. Whole body MBKO mice on the NMRI background [13, 80] where backcrossed on the C57BL/6N background for more than six generations. MBKO mice showed a similar phenotype in response to diet‐induced obesity as previously described (Figure 9A–C and Figure S5A–C).

Mice were bred at the Sächsische Inkubator für Klinische Translation (SIKT), Leipzig. All mice were housed in pathogen‐free facilities at 23°C on a 12 h light/dark cycle, were fed a standard chow diet (V1534, 9 kJ% from fat) or high fat diet (D12492, 60 kJ% from fat) with ad libitum access to food and water, except when indicated. Animals were sacrificed by CO_2_ overdose, and samples were collected and processed as described in the following.

### 4.3 Phenotyping

Male and female WT and ATMBKO mice were studied from an age of 11 up to 24 weeks under chow and HFD conditions. Body weight was recorded weekly and whole‐body fat mass as well as lean body mass were recorded in awake animals using an EchoMRI‐700 instrument (Echo Medical Systems, Houston, TX, USA) at an age of 10, 16 and 24 weeks. Glucose and insulin tolerance tests (GTT and ITT) were conducted after 18 and 19 weeks after the diet started. For the GTT, mice were fasted for 6 h before i.p. application of glucose solution (2 g/kg body weight, 20% glucose solution). Blood sugar was measured before application and after 15, 30, 60, and 120 min. For the ITT, mice were fasted 2 h before i.p. injection of insulin (Insuman Rapid 100; 0.75 U/kg body weight under chow diet; 1.5 U/kg body weight under HFD). Blood sugar was measured before application and after 15, 30, and 60 min. Blood samples for measurement were taken from the tail vein using the FreeStyle Mini automated glucose monitor (Abbott GmbH, Ludwigshafen, Germany). Fasted animals had free access to water.

### 4.4 Cold Exposure

For cold exposure experiments, mice at 12 weeks of age were adapted to single housing (23°C) in rodent climate chambers (MKKL1200) for at least 3 days. Mice were transferred into a climate chamber set to 8°C and remained there for 7 days. Thermal images were done as described in 4.2.4 at the beginning, after 6 h and at the end of the experiment. Rectal body temperature was measured hourly in the first 6 h, as well as every day at 8 am.

### 4.5 Fasting‐Feeding‐Transition

For fasting experiments, mice at 12 (chow) or 24 (HFD) weeks of age were adapted to single housing (23°C) in rodent climate chambers (MKKL1200). Food restriction was carried out 1 h before the start of the night phase (05:00 pm) and mice were refed 16 h after food withdrawal. Rectal body temperature, body weight, and blood glucose measurements were done before and after fasting, as well as 8 h after refeeding.

### 4.6 Temperature Measurements and Indirect Calorimetry

Body temperature measurements using a special probe (TH‐5 Thermalert Clinical Monitoring Thermometer, Physitemp Instruments). Whole body energy metabolism was analyzed with an indirect calorimetry metabolic chamber system (Phenomaster) at an age of 12 (chow) or 22 (HFD) weeks. Mice were adapted to single housing (23°C) in rodent climate chambers (MKKL1200) for at least 3 days before transferring to CaloSys V2.1 metabolic chambers (TSE Systems, Bad Homburg, Germany). To measure energy expenditure dependent on ambient temperature, various parameters (e.g., VO2, VCO2, energy expenditure, locomotor activity) were recorded every 5 min for 5 days at 23°C (mild cold stress), followed by 5 days at 30°C (thermoneutrality, inactive BAT) and finally 5 days at 8°C (cold stress, highest BAT activity). Indirect calorimetry data was analyzed using CalR Version 2 [81].

### 4.7 AT Histology and Immunofluorescence

For AT histology, measurements of lipid droplet and adipocyte size distributions as well as immunohistochemical analyses were performed. iWAT and gonadal WAT as well as interscapular BAT were collected and fixed in paraformaldehyde (4%, pH 7.4) for 24 h at 4°C. After paraffin embedding and sectioning, tissues were stained with hematoxylin and eosin. Five images of HE stained WAT sections were taken per sample at 200x magnification with the Keyence BZ‐X800 (Keyence Corp., Osaka, Japan). Some of the images were loaded as a training set into the freely available Ilastik software [82] and used to train a pixel‐based classifier. The trained classifier was used to analyze the entire data set. The calculated probability maps were output as a png document. The microscopic images and the probability maps calculated for each image were loaded into the open‐source software CellProfiler 4 [83] and the adipocytes were identified and measured using automated analysis.

Immunohistochemistry was done using rabbit anti‐UCP1 polyclonal antibody (ab10983, Abcam, Cambridge, UK), anti‐MB antibody (ab77232), and HRP‐conjugated anti‐rabbit antibody (Dako Envision+; Dako, Jena, Germany) and images were taken using a Keyence BZ‐X800 fluorescence microscope (Keyence Deutschland GmbH, Frankfurt am Main, Deutschland). Immunofluorescence was done using anti‐UCP1 polyclonal antibody (ab10983, Abcam, Cambridge, UK), monoclonal anti‐MB antibody (sc‐393020, Santa Cruz, Dallas, TX, USA), monoclonal anti‐PLIN2 (ab108323, Abcam), Wheat Germ Agglutinin (WGA) Alexa Fluor 488 (Thermo Fisher, #W11261, Waltham, MA, USA)and DAPI (Thermo Fisher, #D21490, Waltham, MA, USA). Images were taken using ZEISS LSM980 Airyscan 2 (Carl Zeiss, Jena, Germany).

### 4.8 Electron Microscopy

For electron microscopy, BAT was prepared into 1 mm^3^ pieces using a razor blade. BAT pieces were fixed in paraformaldehyde (4%) and glutaraldehyde (2%) in phosphate buffered saline (PBS) for 24 h. Following fixation, the samples were stained with osmium tetroxide (0.5%; EMS, Hatfield, PA, USA) in PBS. After thorough rinsing, the tissue was dehydrated in graded alcohol and further stained with uranyl acetate (1%; Merck, Darmstadt, Germany) in alcohol (70%). After further dehydration, the samples were transferred in propylene oxide (Sigma–Aldrich, Steinheim, Germany) and incubated in Durcupan (Sigma–Aldrich). After polymerization at 56°C for 48 h, the blocks of resin were trimmed and finally cut using an ultra‐microtome (Leica Microsystems, Wetzlar, Germany). Ultra‐thin sections with an average thickness of 55 nm were transferred on formvar‐coated copper grids and stained with lead citrate. Analysis was performed using a Zeiss SIGMA electron microscope (Zeiss NTS, Oberkochen, Germany) equipped with a STEM detector and ATLAS software. Images were acquired at an original magnification of 12 000x and a pixel size of 2.3 nm. Mitochondria were analyzed and measured using shape descriptors of ImageJ v1.53.

### 4.9 Bulk RNA Sequencing

RNA isolation from AT was done using QIAzol lysis reagent and the RNeasy Lipid Tissue Mini kit (Qiagen, Hilden, Germany) as specified by the manufacturer. The quality control of total RNA was done with the Fragment Analyzer 5200 (Agilent) using the High Sensitivity RNA quantification kit and Fragment Analyzer Controller Software (Agilent v3.1.0.12). Random primed library preparation was started with 150 ng of total RNA using the Watchmaker RNA library prep kit with Polaris depletion (Watchmaker Genomics) according to the instructions of the manufacturer. The barcoded libraries were purified and quantified using Qubit Fluorometric Quantification (ThermoFischer Scientific). Size distribution of the library DNA was analyzed using the Fragment Analyzer 5200 (Agilent). Sequencing of 2×150 bp was performed with an Illumina NovaSeq 6000 sequencer (Illumina, San Diego, CA, USA) at the sequencing core facility of the Faculty of Medicine (University Leipzig) according to the instructions of the manufacturer. Sequences were demultiplexed with bcl2fastq software (Illumina, v2.20).

Raw RNA‐seq reads were subjected to quality control and adapter removal with Trimmomatic (v0.39) [84], and reads shorter than 18 bp were discarded. The filtered reads were then mapped to the GRCm38.p6 mouse reference genome using STAR (v2.7.8a) [85] with a limit of up to 50 permissible multi‐mapped alignments. Gene‐level read counts were obtained with featureCounts from the Subread package (v2.0.1) [86], assigning multi‐mapped reads proportionally. Data were normalized using the variance stabilizing transformation (VST). Differential gene expression (DGE) analysis was carried out with DESeq2 (v1.42.1) [87], and genes with a false discovery rate (FDR) < 0.05 and an absolute log_2_ fold change (FC) ≥ 1 were classified as significantly differentially expressed. To avoid artifacts associated with the *Adipoq‐Cre* insertion site [88], all passenger genes co‐integrated with the transgenic construct were removed prior to downstream analysis.

Gene set enrichment analysis (GSEA) [89, 90] was performed on bulk RNA sequencing data using the GSEA software (Broad Institute, v4.3.3). A ranked gene list containing all measured transcripts was generated (rank score = log_2_FC × −log_10_(adj. p‐value)). This ranked list was used as input for GSEA analysis against the hallmark [91] gene sets from the Molecular Signatures Database (MSigDB, v2025.1). Normalized enrichment scores and false discovery rate–corrected q‐values were used to determine significantly enriched pathways. Pathways with FDR q < 0.05 were considered as significantly enriched.

To quantify contractile (muscle‐enriched) brown adipocyte activity in bulk BAT RNA‐seq, we calculated an expression score based on marker genes of the contractile adipocyte subcluster identified in single nuclei RNA sequencing data of BAT [41]. Marker genes with adjusted *p* < 0.05 and an enrichment (cluster expression difference) > 0.1 were retained. For each sample, the GSVA R package (v2.2.1, [92]) was used to compute the enrichment score representing the relative expression of the retained marker gene set against the transcriptome‐wide background.

### 4.10 Targeted Metabolomics

Arterial blood was collected, and serum was separated immediately after sampling. To preserve metabolic integrity, samples were rapidly stabilized and stored at ultra‐low temperature until analysis. Targeted metabolomics profiling was performed using the MxP Quant 500 Kit (Biocrates Life Sciences AG), which enables absolute quantification and high‐confidence annotation of a broad panel of metabolites across multiple biochemical classes. Sample preparation and measurement were carried out following the manufacturer's standardized workflow using liquid chromatography‐tandem mass spectrometry (LC‐MS/MS) and flow injection analysis–tandem mass spectrometry (FIA‐MS/MS). Metabolite identification and quantification were based on isotope‐labeled internal standards and a calibrated reference standard curve provided within the kit. Data acquisition and processing were performed using Biocrates MetIDQ software, including automated quality control, normalization, and validation against predefined technical criteria. Only metabolites meeting internal quality thresholds were included in statistical analysis. Low‐quality and infrequently detected metabolites in less than 90% of samples were removed. Missing values were subsequently imputed using a k‐nearest neighbors approach, assuming that samples with similar overall metabolic profiles exhibit comparable metabolite intensities using the R package pmp (v1.20.0 https://bioconductor.org/packages/release/bioc/html/pmp.html). Metabolite intensities were log_10_‐transformed and standardized by z‐score normalization to ensure comparability of dynamic ranges. Downstream statistical analysis was done using MetaboAnalyst 6.0 [93].

### 4.11 Untargeted Proteomics

For proteomic analysis, 5 µg total protein of each lysate was reduced, alkylated, and digested with trypsin using a paramagnetic bead–based workflow (Cytiva, USA). Peptides were analyzed by LC‐MS/MS using a Vanquish Neo UHPLC (Thermo Fisher Scientific) coupled to an Orbitrap Astral mass spectrometer. For each run, 400 ng of peptides were loaded onto a trap column PepMap Neo Trap (0.5 cm x 300 µm, 5 µm, 100 Å, C18; Thermo Fisher Scientific) and separated on an analytical column AUR3‐25075C18‐XT (25 cm × 75 µm, 1.8 µm, 120 Å, C18; IonOpticks) maintained at 50°C. Peptides were eluted at 0.4 µL min^−^^1^ over a 30 min three‐step gradient using solvent A (formic acid (0.1%) in water) and solvent B (acetonitrile (80%), formic acid (0.1%)) with step 1. 0.40–16.5 min: 8% to 25% B, step 2: 16.5–22.5 min: 25% to 35% B and step 3: 22.5–26.0 min: 35% to 48% B.

Column washing and re‐equilibration were performed at 0.5 µL min^−^^1^ and completed with 99% B (26.0–30.0 min). The electrospray was operated in positive‐ion mode at 1.5 kV with the ion transfer tube at 280°C. The Orbitrap Astral was operated in data‐independent acquisition (DIA) mode. Full MS scans were acquired at 240 000 resolution over m/z 380–980, with one MS1 survey scan every 0.6 s. DIA windows covered m/z 380–980 using automatically generated, non‐overlapping 2 m/z isolation windows (299 total). MS2 spectra were generated using HCD with 25% normalized collision energy and detected on the Astral over m/z 150–2000. The maximum injection time was 3 ms and the AGC target was 500% for both MS1 and MS2 scans.

Data processing protocol: A spectral library was generated in silico using DIA‐NN 2.0 with default settings, based on the UniProtKB Mus musculus reference proteome (reviewed entries and isoforms; 08‐15‐2025). Raw DIA data were processed using DIA‐NN 2.0 with default parameters. Peptide and protein quantifications were imported into Perseus 1.6.2.3. Downstream data processing, including filtering, median normalization, variance stabilization, and missing‐value imputation, was performed using the proteomicsr R package (https://zenodo.org/records/10171433, [94]). Differential protein abundance analyses were conducted using the same workflow. Gene set enrichment analysis (GSEA) [89, 90] was performed on untargeted proteomics data using the GSEA software (Broad Institute, v4.3.3). A ranked gene list containing all measured transcripts was generated (rank score = log_2_FC × −log_10_(p‐value)). This ranked list was used as input for GSEA analysis against the hallmark [91] gene sets from the Molecular Signatures Database (MSigDB, v2025.1). Normalized enrichment scores and false discovery rate‐corrected q‐values were used to determine significantly enriched pathways. Pathways with FDR *q* < 0.05 were considered as significantly enriched.

### 4.12 In Vivo Transfection

After twelve weeks on the high fat diet, whole body MBKO mice were randomized according to their body weight into the treatment groups (vehicle *n* = 4; hMB *n* = 5). In vivo transfection of mammalian expression vector pcDNA3.1(+) encoding human MB (hMB group) or empty vector (control group) was performed using linear polyethyleneimine (PEI; in vivo‐jetPEI, Sartorius, Göttingen, Germany) according to the high concentration protocol provided by the manufacturer. Briefly, 20 µg of plasmid DNA were diluted in glucose solution (10%) and sterile water to reach a final glucose concentration of 5%. Then, the appropriate volume of in vivo‐jetPEI was added for a final N/P ratio of 8, mixed, spun down and incubated for 15 min at room temperature. For in vivo‐transfection, 50 µL transfection mix was injected subcutaneously over the interscapular BAT. Injections were repeated after three and seven days. Phenotyping was continued for 21 days after the first application.

### 4.13 In Vitro Experiments

#### 4.13.1 Adipocyte Cell Culture

Primary interscapular brown adipocytes from male C57BL/6N mice, MBKO and WT littermates, as well as immortalized mouse brown adipocytes (imBA [95]) and SGBS cells [96] were cultured and differentiated as previously described [97].

#### 4.13.2 Human Primary Adipocytes

Primary human adipocytes were collected from subcutaneous AT samples collected during elective aesthetic and body contouring surgery after weight loss at the Division of Plastic, Aesthetic and Special Hand Surgery of University Hospital Leipzig. Adipocytes were isolated after mechanic homogenization, enzymatic digestion, and filtration, and mature floating adipocytes were cultivated as membrane mature adipocyte aggregate cultures (MAAC, [98, 99]). Human primary brown adipocytes were obtained, cultured, and differentiated as previously described [100]. Following differentiation, adipocytes were incubated with either vehicle or 10 µm norepinephrine for 4 and 24 h.

#### 4.13.3 Adipocyte Transfection

MB overexpressing imBA were generated by stable transfection using human MB (WT and mutant MB_K45A/F46W/K63A/H64W) encoding pcDNA3.1(+) plasmids (Genscript, Piscataway, NJ, USA) via nucleofection (transfection program CM‐137, 4D‐Nucleofector, Lonza, Basel, Switzerland). Transfected cells were cultured under antibiotic selection.

MB overexpressing primary human mature adipocytes, SGBS and primary murine mature adipocytes were generated by transient transfection using a human MB containing (WT and mutant MB_ K45A/F46W/K63A/H64W) pcDNA3.1(+) plasmids (Genescript, Piscataway, NJ, USA) via electroporation (voltage: 1550 V; pulse width: 20 ms; pulse number: 2; Neon NxT Electroporation System, Thermo Fisher, Waltham, MA, USA). Empty vector‐transfected cells served as controls.

#### 4.13.4 SiRNA‐Mediated Gene Silencing

siRNA‐mediated knockdown was performed based on a recent publication [101]. For reverse siRNA‐mediated gene knockdown, differentiated imBA (day 6) were detached and counted. In the meantime, 5 µL Lipofectamine RNAiMAX per 1 mL Opti‐MEM was mixed and siRNA was diluted in Opti‐MEM to a concentration of 400 nm. Next siRNA and Lipofectamine solution were mixed 1:1 and 25 µL were added to each well of the cell culture plate (Seahorse XF Pro M 96‐well Cell Culture Microplate). Afterwards, 1.25 × 10^4^ differentiated imBA in 75 µL cell culture medium were added in each well to the siRNA‐Lipofectamine solution. Cells were incubated for 48 h until day 8, when medium was replaced. Experiments were performed on day 9 of differentiation.

#### 4.13.5 Mitochondrial Respiration Measurements Using the Seahorse Analyzer

Mouse imBA, primary brown adipocytes, as well as human SGBS adipocytes were pre‐differentiated in 6‐well plates until day 6 of differentiation, detached from plates, transfected with siRNA via lipofection or an hMB plasmid via electroporation in the corresponding assays and subsequently re‐seeded into 96‐well Seahorse assay plates. Assays were performed as previously published with minor modifications [99]. Adipocytes were washed with assay medium (glucose (10 mm), L‐glutamine (2 mm), sodium pyruvate (1 mm) in Seahorse XF base medium at pH 7.4) and then incubated in assay medium for 45–60 min at 37°C at low CO_2_. For mitochondrial stress tests, OCRs were measured in a Seahorse XF Pro analyzer (Agilent Technologies, Santa Clara, CA, USA) with the following injections: oligomycin (2 µm, Oligo), FCCP (2 µm), and rotenone/antimycin A (1 µm, Rot/AA) (103015‐100, Agilent). For β‐adrenergic or adenylyl cyclase agonist treatment, FSK (1 µm) was acutely injected between Oligo and FCCP injections. For mitochondrial FFA oxidation assays, 1 × 10^4^ primary mouse brown adipocytes or 1.25 × 10^4^ immortalized brown adipocytes were seeded per well at day 6 of differentiation into XF96 plate and incubated with siRNA RNAiMAX as described previously [13]. At day 8, medium was replaced by starvation medium (DMEM with FCS (2%)) for 16 h. At day 9, cells were washed two times with Seahorse XF DMEM medium, pH 7.4 supplemented with glucose (2 mm) and L‐Carnitine (substrate limited assay medium) and then incubated with 150 µL substrate limited assay medium for 1 h at 37°C in an incubator without CO_2_. Immediately before start of the assay, 30 µL BSA‐conjugated fatty acid (BSA‐Oleate, Chayman Chemicals No. 29557; BSA‐Octanoate, Chayman Chemicals No. 34933; BSA‐Palmitate, Chayman Chemicals No. 29558) were added to the cells to a final concentration of 200 µm. OCRs were measured in cycles of 4 min mixing and 2 min measuring in a Seahorse XF Pro Analyzer with the following injections and port loading: A: oligomycin (2 µm), B: etomoxir (5 µm) or substrate limited assay medium, C: FCCP (2.4 µm), D: Rot/AA (0.5 µm). Analysis was performed with Wave Pro software (version 10.1.0, Agilent). To minimize the variability of the oxygen consumption rate, we optimized the seeding to the same cell number and adipogenesis was induced in fully confluent preadipocytes. Therefore, normalization to seeded cell number was performed. Primary human adipocytes were measured after Matrigel embedding as previously described in detail [102].

#### 4.13.6 High Resolution Respirometry Using the OROBOROS O2k Respirometer

Mitochondrial respiration ex vivo analysis was performed in differentiated imBA using the high‐resolution Oxygraph‐2k (OROBOROS Instruments, Innsbruck, Austria) as previously described [103]. Prior, mouse imBA were differentiated until day 6 as described above. At day 6, cells were detached and reverse‐transfected with siRNA as described above, followed by reseeding into 6‐well plates. Cells were maintained under differentiation conditions for an additional 3 days. On day 9, cells were detached, counted, and 1 million cells per replicate were collected and resuspended in mitochondrial respiration medium (Mir05: EGTA (0,5 mm), MgCl2•6H2O (3 mm), K‐lactobionate (60 mm), taurine (20 mm), KH_2_PO_4_ (10 mm), HEPES (20 mm), sucrose (110 mm), and fatty acid‐free BSA (1 g/L)) and immediately transferred to the chambers the respirometer at 37°C. Cells were permeabilized with digitonin (5 µg/mL) and mitochondrial respiratory capacity was analyzed performing a multiple substrate‐uncoupler‐inhibitor titration (SUIT) protocol under normoxic conditions using following substrate concentrations: malate (0.5 mm) + ADP (5 mm) (submaximal CI‐linked respiration), octanoylcarnitine (0.5 mm) (fatty acid oxidation, FAO); cytochrome c (10 µm) (integrity of outer mt‐membrane), pyruvate (5 mm) + glutamate (10 mm) + malate (2 mm) (maximal CI‐linked respiration), succinate (10 mm) (maximal CI‐ and CII‐linked respiration, maximal OXPHOS), rotenone (0.5 µm) + antimycin A (2.5 µm) (CI and CIII inhibition, residual oxygen consumption, ROX), tetramethyl‐p‐phenylendiamin (TMDP, 0.5 mm) (CIV activity assay) + ascorbate (2 mm), sodium azide (100 mm) (CIV inhibition, chemical oxygen background). OCR was normalized to cell number and corrected for ROX (after CI and CIII inhibition) and chemical oxygen background (after CIV inhibition).

#### 4.13.7 Lipolysis Assay

NEFA concentration was measured from supernatant of differentiated adipocytes (imAB, primary brown, SGBS) under basal conditions and adrenergic stimulation with CL (1 µm) or FSK (1 µm) for 1 h in lipolysis assay reagent (FFA free BSA (2% w/v) in DMEM) with the NEFA‐HR(2) assay kit (Fujifilm Wako Chemicals Europe GmbH, Neuss, Germany). For transfected human mature adipocytes, measurements were done under basal conditions and adrenergic stimulation with isoproterenol (1 µm) for 1.5 h in lipolysis assay reagent (FFA free BSA (2% w/v) and glucose (2 mm) in KRBB). At the end of the experiment, 10 µL of the supernatant was mixed with 150 µL R1 and incubated for 3 min at 37°C. Afterwards, 75 µL R2 was added and incubated for 8 min at 37°C. Absorbance was measured at 546 and 660 nm for reference.

#### 4.13.8 Fatty Acid Uptake Assay

Fatty acid uptake was measured with the QBT Fatty acid uptake assay (Molecular Devices, San Jose, CA, USA) according to the manufactures protocol. Differentiated adipocytes in a 96‐well plate were starved for 1 h in 90 µL serum free DMEM at 37°C, 5% CO_2_. For measuring basal conditions, 10 µL 1x HBSS (without BSA, pH 7.4) or 10 µL of CL (1 µm) in 1x HBSS (without BSA, pH 7.4) were added. Immediately 1× Loading Buffer (Component A with 1x HBSS with FFA free BSA (0.2% w/v)) was added to the samples, followed by fluorescence measurement. Excitation was set to 470–500 nm, and emission was recorded at 500–560 nm. Kinetic readings were performed every 20 s over 90 min.

#### 4.13.9 Gene Expression Analysis

RNA isolation from ATs and adipocytes (primary adipocytes, imBA, SGBS) was done using the RNeasy Lipid Tissue Mini kit. Quantitative real‐time PCR was performed using the LightCycler System LC480 and LightCycler‐DNA Master SYBR Green I Kit (Roche, Basel, Switzerland). Gene expression was calculated by the ΔΔCT method and normalized to *36b4* or *Nono* levels in each sample, as indicated. For human primary brown adipocytes, RNA was isolated and qPCR performed as previously described [100], except for *UCP1* and *PPIA* mRNA was measured using UPL probes. Transcript levels are presented as the ratio of the abundance of the gene of interest to the mean abundance of the control genes *RNA18S5* and *PPIA*. Primer sequences are listed in Table S9.

#### 4.13.10 SDS‐PAGE, Western Blot and Capillary‐Based Immunoassay

Proteins from cells or tissues were extracted with RIPA buffer (NaCl (150 mm), Tris (10 mm, pH 7.2), SDS (0.1%), TX100 (1%), deoxycholate (1%), EDTA (5 mm); supplemented with protease inhibitor cocktail (11697498001, Roche) and phosphatase inhibitor cocktail (#4906845001, Roche). Protein expression was analyzed using the Jess automated capillary‐based immunoassay system (ProteinSimple, San Jose, CA, USA) or by classical Western blot.

For capillary‐based immunoassay, samples were run using a 12–230 kDa separation module and a 25‐capillary plate format with integrated Total Protein Normalization. Anti‐MB primary antibody (Santa Cruz Biotechnology #393020) was detected using the manufacturer's anti‐mouse Simple Western detection reagents. Data were acquired and quantified using Compass software (ProteinSimple).

For Western blot, samples were subjected to SDS‐PAGE and transferred to nitrocellulose membranes using the tank blot method as previously described [104]. After incubation (overnight at 4°C) with primary antibodies, HRP‐coupled secondary antibodies were used and chemiluminescence was detected and quantified using the G:BOX Chemi XX9 system with GeneSys and GeneTools software (SynGene, Bengaluru, Karnataka, India). The following antibodies were used: from Cell Signaling Technologies: phospho‐PKA substrates (RRXS^*^/T^*^) (#9624), TUB1A (#2144), FASN (#3189S), AKT (#C67E7), pAKT (#S473), HSL (#4107S), pHSL (4126S) mTOR (#2983), p‐mTOR (#2974S), AMPKa (#5831T), pAMPKa (#2535S), anti‐rabbit‐HRP (#7074), anti‐mouse‐HRP (#7076); from Abcam: UCP1 (ab10983); from Sigma‐Aldrich: ACTB (#A1978); from Thermo Fisher Scientific: OXPHOS (#45‐8099); from Santa Cruz Biotechnology: MB (#393020)

#### 4.13.11 ELISA

MB expression in BAT lysates of in vivo transfected mice was analyzed by ELISA according to the manufacturer's protocols (Myoglobin SimpleStep ELISA Kit, ab210965, Abcam).

### 4.14 Statistical Analyses

Data are presented as means ± SEM. Statistical analyses were performed using GraphPad Prism 11, CalR and MetaboAnalyst. Methods of statistical analyses and post‐tests were chosen based on the design of each experiment and are stated in the figure legends. If not stated otherwise, adj. *p* < 0.05 were considered statistically significant.

## Author Contributions

C.S., J.W. and J.T.H. conceptualized, initiated and developed the project. C.S. and J.T.H. wrote the manuscript. C.S., H.B., C.G., R.Z., K.S., M.O., M.K., L.R., K.K., N.K. and J.W. performed or assisted with experiments and analyzed data. T.H., S.M. and A.H. performed bioinformatic analyses. K.S. performed proteomic analyses. V.K. and A.G. contributed and analyzed BAT single nuclei RNAseq data. M.K. and M.W. provided SGBS cells. R.N. provided human adipose tissue samples. M.S., M.B. and R.H.S. contributed human data. J.T.H. coordinated and supervised the project and acquired funding. All authors read, edited, and approved the manuscript.

## Conflicts of Interest

MB received honoraria as a consultant and speaker from Amgen, AstraZeneca, Bayer, Boehringer‐Ingelheim, Lilly, Novo Nordisk, Novartis, and Sanofi. All other authors declare no conflicts of interest.

## Supporting information




**Supporting File 1**: advs76191‐sup‐0001‐SuppMat.pdf.

**Supporting File 2**: advs76191‐sup‐0002‐TableS1‐S9.xlsx.

## Data Availability

All the necessary data to evaluate the conclusions in the paper are provided in the paper itself and/or the Supplementary Materials. The RNAseq data have been deposited with links to the BioProject accession number PRJNA1373549 in the NCBI BioProject database (https://www.ncbi.nlm.nih.gov/bioproject/). The mass spectrometry proteomics data have been deposited to the ProteomeXchange Consortium via the PRIDE [105] partner repository with the dataset identifier PXD076825. The metabolomics data have not been deposited in a public repository, however interested individuals can request access to this data from the corresponding author.
