# Supplementary material for: Adipocyte Myoglobin Is a Determinant of Energy Expenditure and a Potential Target to Limit Obesity
Source: Adv Sci (Weinh). 2026 Jun 25;13(51):e76191. doi: 10.1002/advs.76191 (PMC13335111; doi:10.1002/advs.76191)
Supplement: Supplementary file 1 [file ADVS-13-e76191-s002.pdf]

Supplementary Information

Adipocyte Myoglobin Is a Determinant of Energy Expenditure and a Potential Target to Limit Obesity

Strehlau C, Broghammer H, Gebhardt C, Hoffmann A, Hagemann T, Midilli S, Zimmer R Schubert K, Karagiannakou V, Georgiadi A, Ost M, Krueger M, Roth L, Krause K, Klöting N, Keller M, Wabitsch M, Nuwayhid R, Stimson RH, Stumvoll M, Blüher M, Weiner J, Heiker JT

Supplementary Figures

Supplementary Figure 1

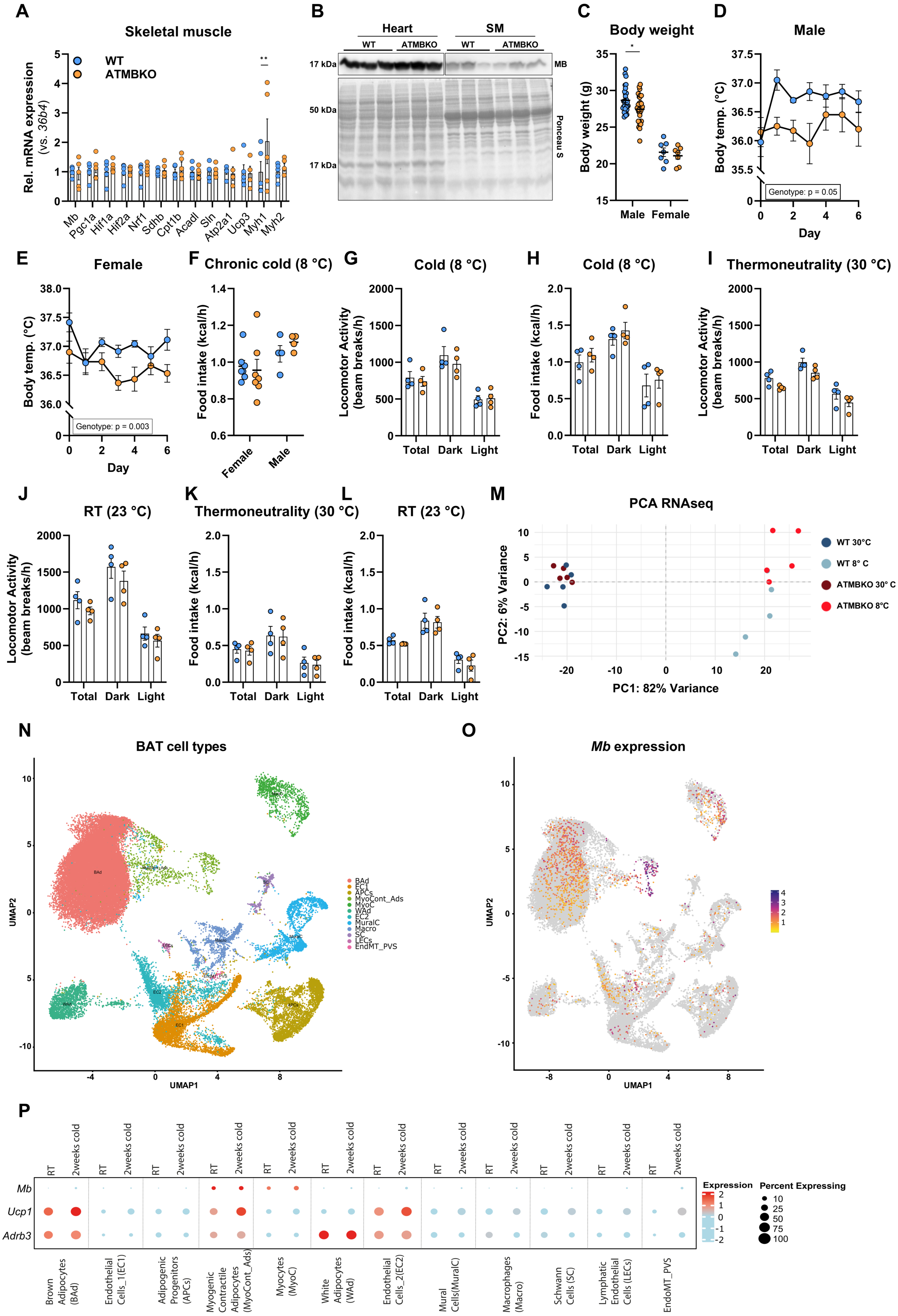

Supplementary Figure 1. (A) mRNA expression of *Mb* and genes involved in oxidative metabolism, hypoxia and muscle thermogenesis (n = 5 per genotype) in the skeletal muscle (quadriceps) from chow-fed WT and ATMBKO mice after 5 days of cold exposure. Gene expression is normalized to *36b4* and relative to WT littermates. **(B)** Representative Western blot detecting MB protein levels in heart and skeletal muscle (SM) from female WT and ATMBKO mice (n = 3/3). **(C)** Body weight of male and female WT and ATMBKO animal at 12 ±1 week depicting age for animal experiments. **(D-E)** Rectal body temperature of male **(D)** and female **(E)** WT and ATMBKO mice exposed to cold conditions (8 °C) for 1 week (n = 7/6 for female, n = 4/4 for male). **(F)** Average food intake of male and female WT and ATMBKO mice exposed to cold conditions (8 °C) for 1 week (n = 7/6 for female, n = 4/4 for male). **(G-L)** Phenotypic characterization from metabolic chambers of male mice. Data represent the average over 3 consecutive days (n = 4/4). **(G)** Locomotor activity and **(H)** food intake per period in cold exposed (8 °C) mice. **(I-J)** Locomotor activity per period in mice housed at **(I)** thermoneutrality (30 °C) and **(J)** room temperature (RT, 23 °C). **(K-L)** Food intake per period in mice housed at **(K)** thermoneutrality (30 °C) and **(L)** RT (23 °C). **(M)** PCA Plot for transcriptome data from BAT of ATMBKO and WT mice housed at thermoneutrality or exposed to cold for 24 h. **(N)** UMAP of single nuclei RNA sequencing data from BAT of WT mice housed at RT as well as chronic cold (14 days) depicting different cell types within the BAT [1]. **(O)** UMAP of single nuclei RNA sequencing data depicting *Mb* expression in different cell types within BAT. **(P)** *Mb* expression in different cell types of the BAT determined by single nuclei RNA sequencing of BAT from WT mice. Data are shown as mean ± SEM. Statistical significance is indicated by asterisks (∗$p < 0.05$, ∗∗$p < 0.01$, ∗∗∗$p < 0.001$) and was determined by two-way ANOVA with Šidák post hoc test (A, D, E) and Students t-test (C).

Supplementary Figure 2

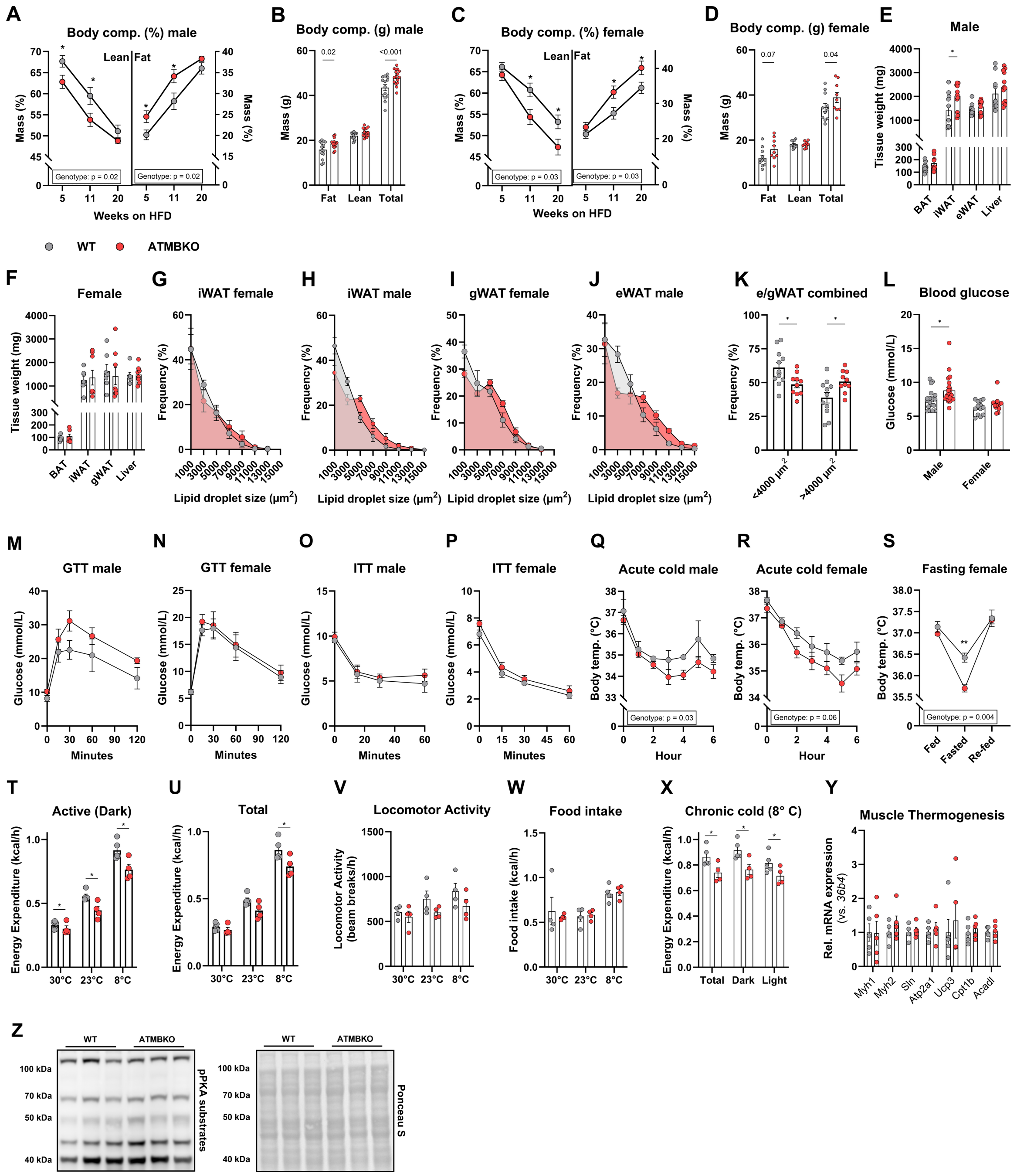

Supplementary Figure 2. (A) Percentage of lean and fat mass after 5, 11, and 20 weeks on a HFD and **(B)** total lean and fat mass after 20 weeks on a HFD in male WT and ATMBKO mice as determined by EchoMRI after 5, 11, and 20 weeks on a HFD (n = 18/19). **(C)** Percentage of lean and fat mass after 5, 11, and 20 weeks on a HFD and **(D)** total lean and fat mass after 20 weeks on a HFD in female WT and ATMBKO mice as determined by EchoMRI (n = 11/9). **(E-F)** Absolute tissue weights after 20 weeks of HFD-feeding in **(E)** male and **(F)** female ATMBKO and WT mice (n = 9/7 for female, n = 11/12 for male). **(L)** Blood glucose concentration after 20 weeks of HFD-feeding in male and female ATMBKO and WT mice (n = 9/7 for female, n = 11/12 for male). **(M–N)** Intraperitoneal glucose and **(O-P)** insulin tolerance tests in male and female HFD-fed WT and ATMBKO mice (GTT: n = 8/6 for female, n = 5/5 for male; ITT: n = 9/7 for female, n =

5/5 for male). **(Q-R)** Male and female WT and ATMBKO mice on HFD were fasted (2 h pre cold exposure) and exposed to acute cold (8 °C) (n = 5/4 for female, n = 3/5 for male). Body temperature of male **(P)** and female **(Q)** ATMBKO and WT mice was measured. **(S)** Body temperature of female (1) fed, (2) fasted overnight for 16 h and (3) re-fed for 8 h (n=5). **(T-X)** Male WT and ATMBKO mice housed in metabolic cages at RT (23 °C), thermoneutrality (30 °C), and chronic cold (8 °C). Data represent the average over 3 consecutive days (n = 5/4). Bar graphs showing energy expenditure for **(T)** nighttime and **(U)** full day at thermoneutrality, room temperature and during cold. **(V)** Locomotor activity and **(W)** food intake at respective temperatures. **(X)** Energy expenditure for full day, night and day during cold exposure. **(Y)** mRNA expression of genes involved in muscle thermogenesis (n = 5 per genotype) in BAT from chow-fed WT and ATMBKO mice after 5 days of cold exposure. Gene expression is normalized to *36b4* and relative to WT littermates. **(Z)** Representative Western blot analysis of pPKA substrates in BAT of HFD-fed WT and ATMBKO littermates after 5 days of cold exposure. Data are shown as mean ± SEM. Statistical significance is indicated by asterisks ($*p < 0.05$, $**p < 0.01$, $***p < 0.001$) and was determined by two-way ANOVA with Šidák post hoc test (A-D, K, Q-S), Students t-test (L) or by ANCOVA performed in CalR (T-U, X).

Supplementary Figure 3

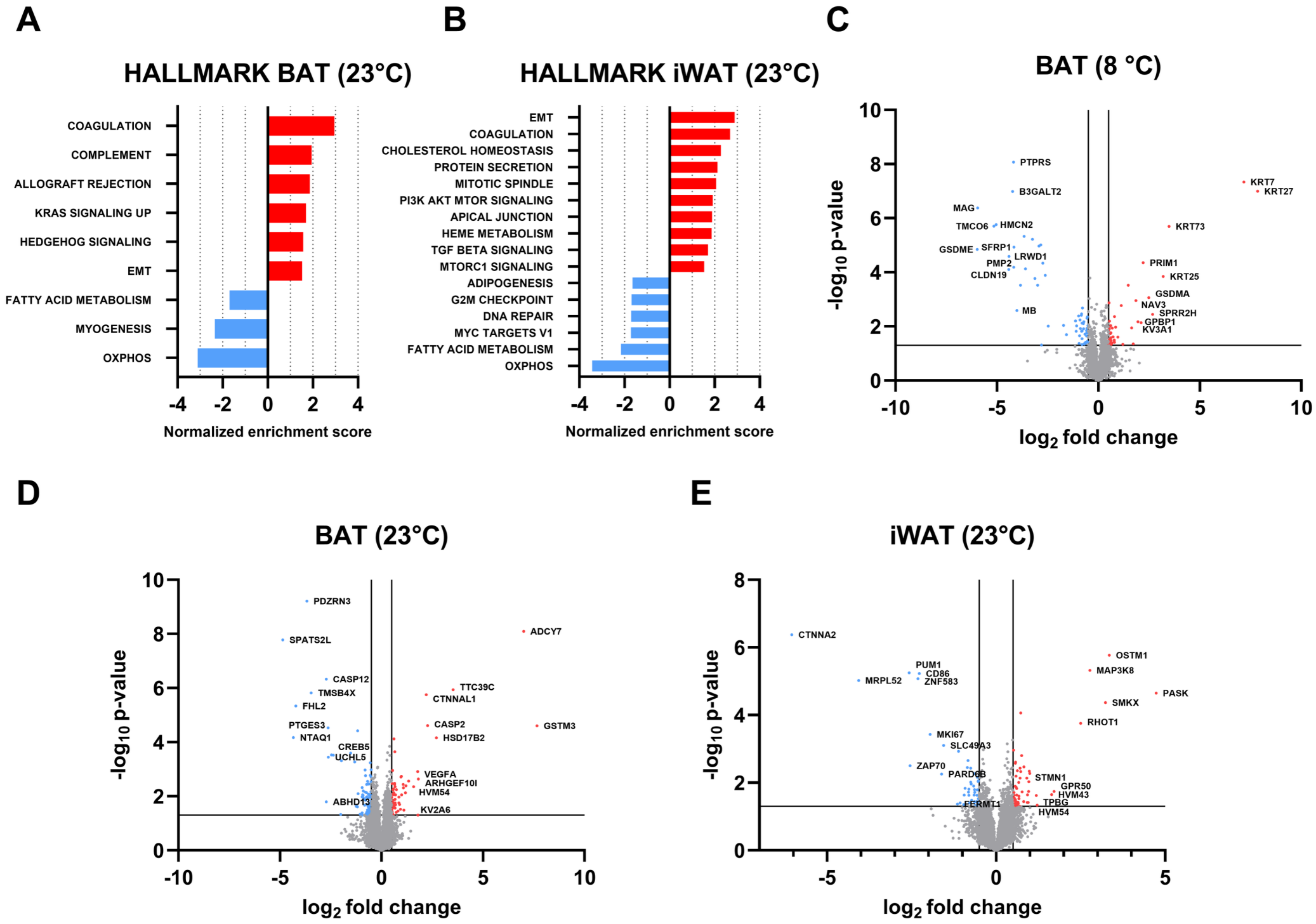

Supplementary Figure 3. (A-B) GSEA of proteomics data from **(A)** BAT and **(B)** iWAT of female HFD-fed WT and ATMBKO mice housed at room temperature (23 °C) and after 20 weeks of HFD. Depicted are all significantly regulated pathways from the Hallmark gene set. **(C-E)** Volcano plots showing significantly regulated proteins in **(C)** BAT of male HFD-fed ATMBKO mice and WT littermates exposed to cold (8 °C) for 5 days and **(D)** BAT and **(E)** iWAT of HFD-fed ATMBKO mice and WT littermates housed at RT (23 °C). Regulation is depicted as ATMBKO compared to WT. Statistical significance for pathway enrichment was determined by Gene Set Enrichment Analysis (GSEA, Broad Institute, UC San Diego) and pathways with q-value ≤ 0.05 were considered significantly regulated.

Supplementary Figure 4

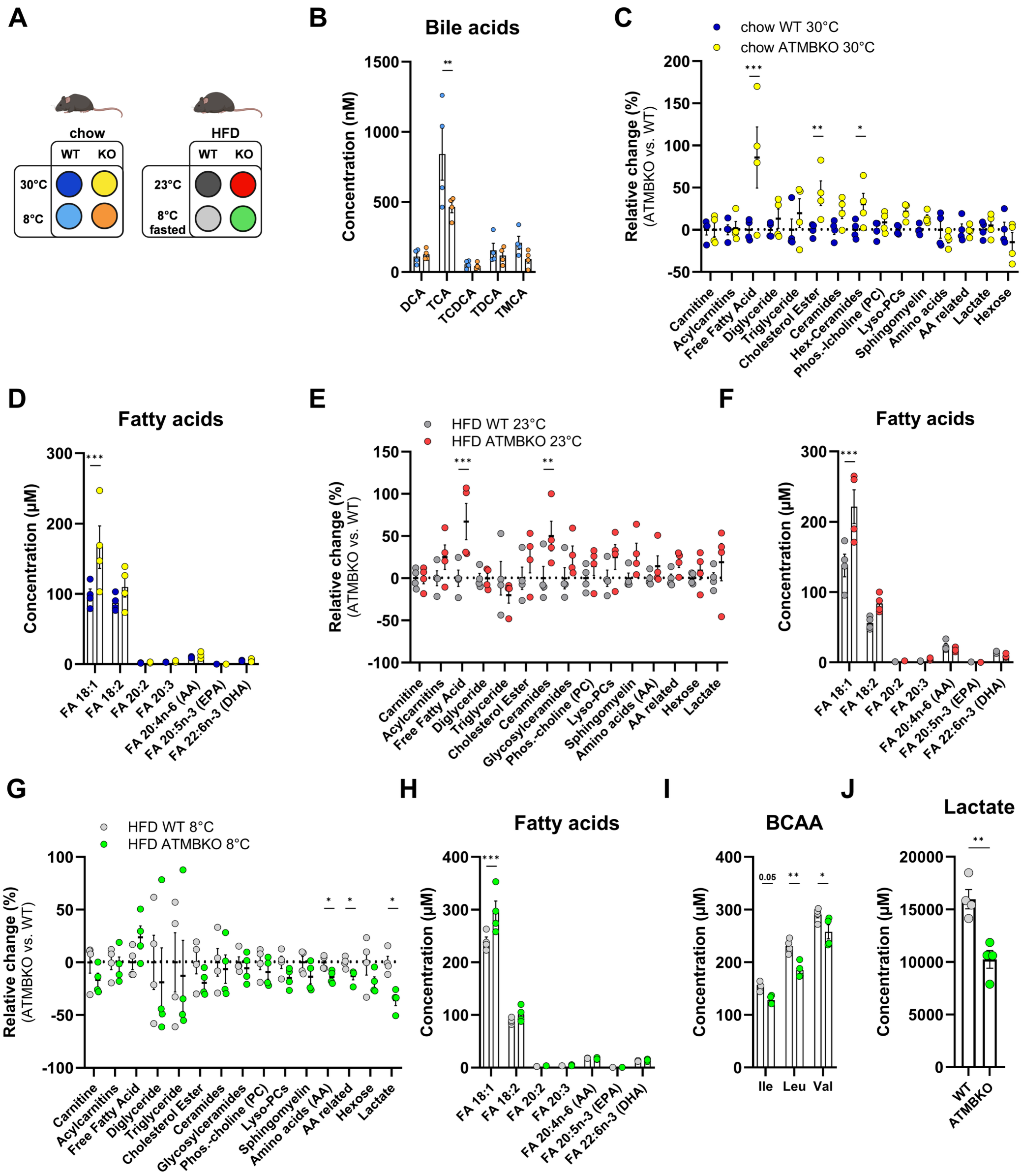

Supplementary Figure 4. (A) Graphic depicting colors of different analyzed groups. **(B)** Absolute concentration of bile acids of chow fed ATMBKO mice vs. WT littermates exposed to cold (8 °C) for 24 h. **(C)** Relative abundance of measured metabolite classes and **(D)** absolute concentration of all measured free fatty acids of chow fed ATMBKO mice vs. WT littermates housed at thermoneutrality (30 °C, 24 h). **(E)** Relative abundance of measured metabolite classes and **(F)** absolute concentration of all measured free fatty acids of chow fed ATMBKO mice vs. WT littermates housed at room temperature and fed ad libitum. **(G)** Relative abundance of measured metabolite classes and absolute concentration of **(H)** all measured free fatty acids, **(I)** BCAA isoleucine, leucine and valine and **(J)** Lactate of chow fed ATMBKO mice vs. WT littermates housed at exposed to short-term cold without access to food (8 °C, 6 h). n = 4 per genotype, data are shown as mean ± SEM. Statistical significance is indicated by asterisks (∗$p < 0.05$, ∗∗$p < 0.01$, ∗∗∗$p < 0.001$) and was determined by two-way ANOVA with Fisher's LSD test (B-I) or Students t-test (J).

Supplementary Figure 5

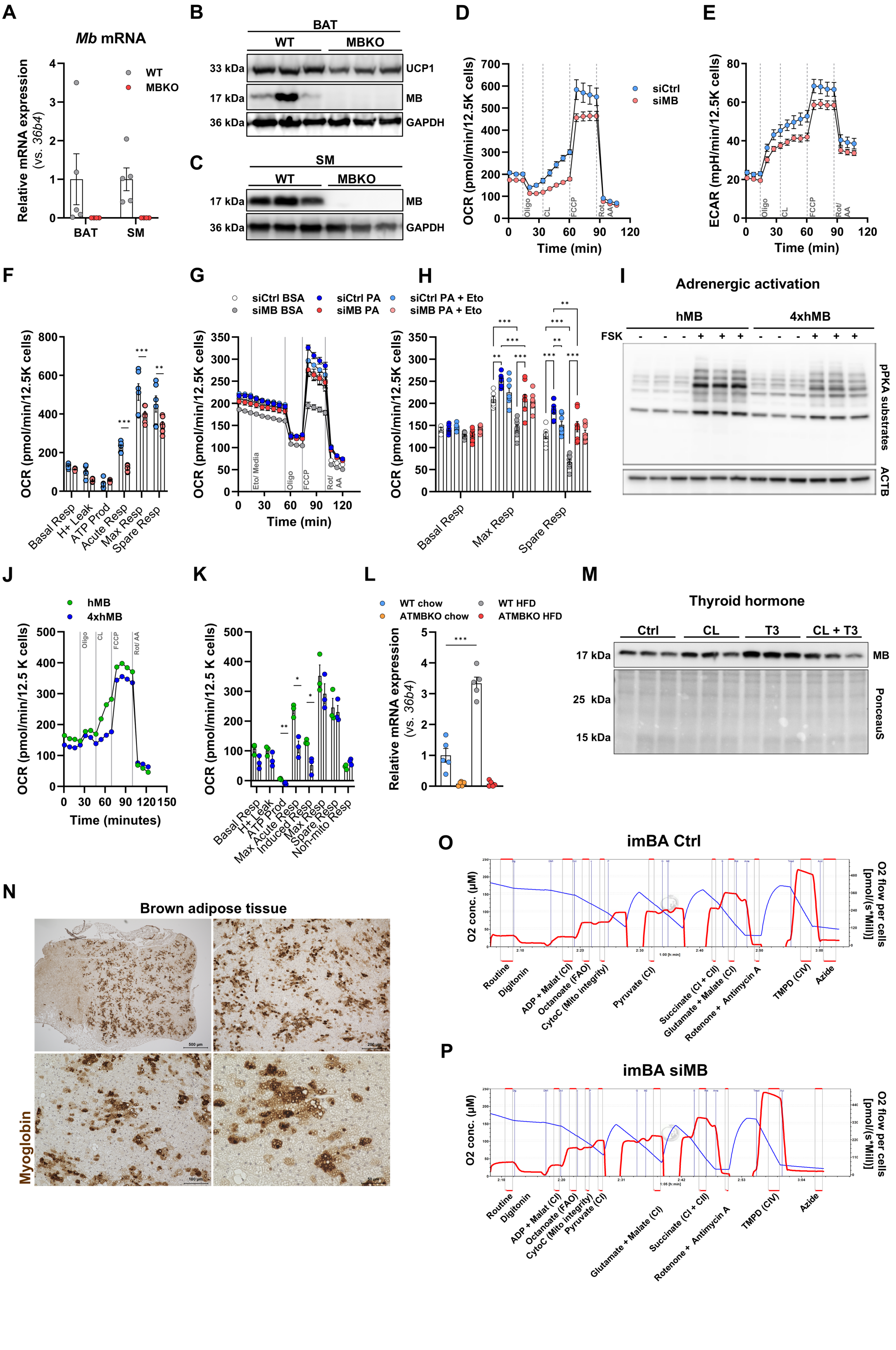

Supplementary Figure 5. (A) Relative Mb gene expression in BAT from HFD-fed male wildtype (WT) and MBKO mice (n = 5/4). **(B-C)** Representative Western blot detecting MB protein levels in **(B)** brown adipose tissue (BAT) and **(C)** skeletal muscle (SM) from HFD-fed male WT and MBKO mice (n = 5/4). **(D-H)** Seahorse measurements of oxygen consumption rates (OCR) after siRNA-mediated knockdown at day 6 of adipogenesis in immortalized brown adipocytes with subsequent evaluation at day 9. **(D)** OCR and **(E)** ECAR of mitochondrial oxidation stress test with acute CL 316,243 (CL) injection and quantification **(F)** of OCR for basal respiration, proton leak, ATP production, acute respiration, maximal respiration and spare respiratory capacity in Ctrl (siScr) and siMB treated imBA (n = 5). **(G-H)** Seahorse measurements of OCR after siRNA-mediated knockdown at day 6 of adipogenesis in immortalized brown adipocytes with subsequent evaluation at day 9. **(G)** Mitochondrial FFA oxidation stress test for palmitic acid (PA) and **(H)** quantification of OCR for basal respiration, maximal respiration and spare respiratory capacity in siCtrl and siMB-treated imBA (n = 6-8). **(I)** Representative Western blot analysis of phosphorylated PKA substrates in 4xhMB and hMB imBA clones basally and after acute stimulation (100 nM FSK, 15 min). Beta-actin served as a loading control (n = 3 per condition). **(J-K)** Seahorse measurements of oxygen consumption rate (OCR) in fully differentiated 4xhMB and hMB imBA clones. **(J)** Standard mitochondrial stress test with acute injection of CL after Oligomycin. **(K)** Quantification of basal respiration (resp.), proton leak, ATP production, maximal acute respiration, induced respiration, maximal respiration, spare respiratory capacity and non-mitochondrial respiration. **(L)** Normalized expression level of *Mb* in chow and HFD fed WT and ATMBKO mice (n = 5 per group and genotype). **(M)** Representative Western blot analysis of MB and UCP1 in imBAs stimulated with CL, T3 and CL + T3 (100 nM; 10 nM) for 24 h and in unstimulated control cells (n = 3 per condition). Ponceau S staining served as a loading control. **(N)** Immunohistochemical staining of MB in BAT of cold exposed WT mice depicting tissue distribution and prominent staining around lipid droplets. Image taken from previous publication with additional magnification [2]. **(O-P)** Representative O_2 flow charts from Oroboros O2k high resolution respirometer depicting O_2 flow per cells and O_2 concentration over time for **(O)** imBA Ctrl (siScr) cells and **(P)** imBA Mb knockdown (siMB) cells. Data are shown as mean ± SEM. Statistical significance is indicated by asterisks (∗p < 0.05, ∗∗p < 0.01, ∗∗∗p < 0.001) and was determined by two-way ANOVA with Šidák post hoc test (C, E, F).

Supplementary Figure 6

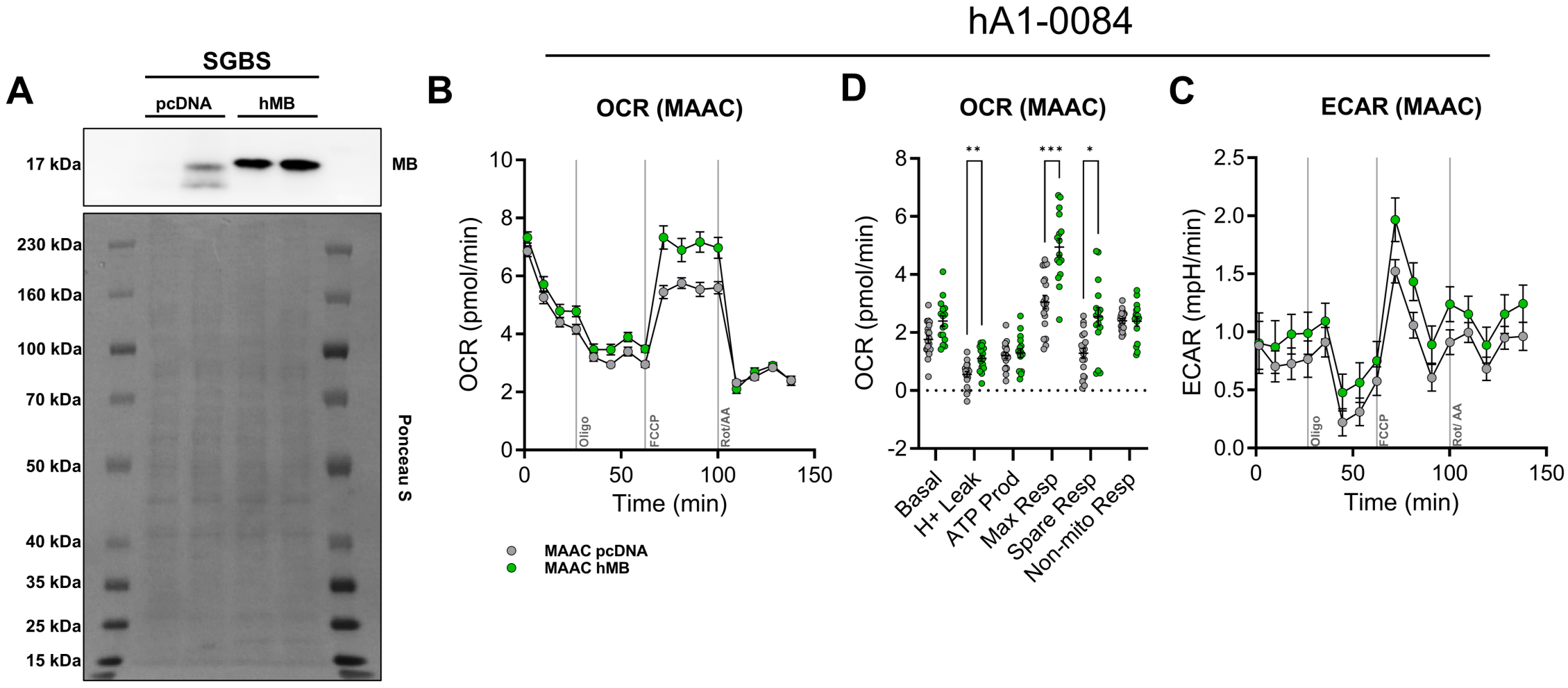

Supplementary Figure 6. (A) Representative Western blot analysis confirming increased MB protein levels after electroporation of hMB plasmid in SGBS cells. **(B-D)** Mitochondrial Stress Test in hMB or pcDNA transfected mature adipocytes (MAAC). **(B)** OCR profile and **(C)** quantification of basal respiration, proton leak, ATP production, maximal respiration, spare respiratory capacity and non-mitochondrial respiration. **(D)** Extracellular acidification rate (ECAR) profile (1 donor, n = 19 for pcDNA/ 16 for hMB). Data are presented as mean ± SEM. Statistical significance is indicated by asterisks (∗p < 0.05, ∗∗p < 0.01, ∗∗∗p < 0.001) and was determined by two-way ANOVA with Šidák post hoc test (C).

References

[1] R. El-Merahbi, V. Karagiannakou, R. Kardinal, L. Seep, R. Lindner, M. Y. Jackstein, S. Hildebrand, M. Hasic, E. Korkmaz, A. K. Jha, A. T. Krokidi, K. Dyar, F. Meissner, S. Grein, J. Heeren, M. Klingenspor, A. Pfeifer, J. Hasenauer, D. Wachten, S. Herzig, A. Georgiadi, *Mol Metab* **2026**, *107*, 102346, https://doi.org/10.1016/j.molmet.2026.102346.

[2] L. Christen, H. Broghammer, I. Rapöhn, K. Möhlis, C. Strehlau, A. Ribas-Latre, C. Gebhardt, L. Roth, K. Krause, K. Landgraf, A. Körner, K. Rohde-Zimmermann, A. Hoffmann, N. Klöting, A. Ghosh, W. Sun, H. Dong, C. Wolfrum, T. Rassaf, U. B. Hendgen-Cotta, M. Stumvoll, M. Blüher, J. T. Heiker, J. Weiner, *Clinical and Translational Medicine* **2022**, *12* (12), e1108, https://doi.org/10.1002/CTM2.1108.
